# Mediterranean diet with high-phenolic EVOO slows kidney function decline and reduces inflammation in nondialysis CKD: a meta-analysis

**DOI:** 10.3389/fnut.2026.1792390

**Published:** 2026-03-02

**Authors:** Cong Zhou, Yutong Li, Manqi Huang, Mingjun Bai, Yanfang Xing

**Affiliations:** 1 Guangdong Provincial Key Laboratory of Major Obstetric Diseases, Department of Nephrology, Guangdong Provincial Clinical Research Center for Obstetrics and Gynecology, The Third Affiliated Hospital, Guangzhou Medical University, Guangzhou, China; 2 The First School of Clinical Medicine, Guangdong Medical University, Zhanjiang, Guangdong, China; 3 Department of Interventional Radiology, The Third Affiliated Hospital of Sun Yat-sen University, Guangzhou, China

**Keywords:** anti-inflammatory, chronic kidney disease, dietary patterns, inflammation, meta-analysis, nondialysis, nutritional intervention, polyphenols

## Abstract

**Background:**

Evidence regarding the renoprotective effect of the Mediterranean diet (MedDiet) in patients with nondialysis chronic kidney disease (CKD) remains inconsistent. This may be partly attributed to variability in the phenolic content of extra virgin olive oil (EVOO), a key bioactive component.

**Methods:**

We conducted a systematic review and meta-analysis to quantify the effect of the MedDiet on renal and cardiometabolic outcomes in adults with nondialysis CKD and to explore the role of high-phenolic EVOO as a potential effect modifier. We searched multiple databases for interventional and observational studies comparing a MedDiet to control diets in adults with CKD stages 1–5. Random-effects meta-analyses were performed.

**Results:**

Ten studies involving 1,073 participants were included. The MedDiet was associated with a modest improvement in estimated glomerular filtration rate (eGFR) (mean difference 2.44 mL/min/1.73 m^2^, 95% CI 0.16 to 4.72), though heterogeneity was high (I^2^ = 90%). This benefit appeared more consistent in mild-to-moderate CKD (eGFR ≥ 45 mL/min/1.73 m^2^). Notably, a significant reduction in C-reactive protein (CRP) was specifically linked to interventions using high-phenolic EVOO (mean difference −0.79 mg/L, 95% CI −1.37 to −0.21). The diet also improved body composition and reduced blood urea nitrogen, without adversely affecting serum potassium or phosphorus. No significant effects were observed on blood pressure or lipid profiles.

**Conclusion:**

In patients with mild-to-moderate CKD, the MedDiet may slow kidney function decline. The benefits appear to be mediated through complementary pathways: renal protection from the overall dietary pattern and a specific anti-inflammatory effect attributable to high-phenolic EVOO.

**Systematic review registration:**

https://www.crd.york.ac.uk/PROSPERO/view/CRD420251124826, identifier PROSPERO (CRD420251124826).

## Introduction

Chronic kidney disease (CKD) is a major global public health burden, affecting over 800 million individuals and conferring high risks of progression to kidney failure and cardiovascular events (1, 2). Medical nutrition therapy is a cornerstone of CKD management (3). Conventional renal diets often emphasize restriction of single nutrients, such as potassium and phosphate, an approach that may compromise overall dietary quality and long-term adherence (4–6). Consequently, there is growing interest in holistic, food-based dietary patterns that support renal health while promoting broader cardiometabolic well-being.

The Mediterranean diet, renowned for its cardiovascular benefits, has emerged as a promising candidate (7). This pattern is characterized by high consumption of plant-based foods, healthy fats (notably olive oil), and fish, with limited intake of red meat and processed foods. A defining component, extra-virgin olive oil (EVOO), is rich in bioactive phenolic compounds. These compounds possess well-documented anti-inflammatory and antioxidant properties in preclinical models, suggesting a potential mechanistic pathway for renal protection (8–10).

Observational studies in CKD populations link higher adherence to a Mediterranean diet with a slower decline in estimated glomerular filtration rate (eGFR) (11, 12). However, evidence from intervention trials remains inconsistent, with several studies failing to confirm significant renoprotective effects (13, 14). Critically, this discrepancy may stem from heterogeneity in study design, participant characteristics, and variations in the specific composition of the dietary intervention. In particular, the phenolic content of the EVOO used, a potential key modifier of the diet’s biological effects, is frequently unreported and rarely standardized across studies.

To address these persistent uncertainties and to clarify the potential role of EVOO as a key effect modifier, we conducted this systematic review and meta-analysis with the following objectives: first, to systematically synthesize and quantify the existing evidence from interventional and observational studies regarding the effects of the Mediterranean diet on kidney function and safety parameters in adults with nondialysis CKD; second, to evaluate its impact on a comprehensive set of cardiometabolic, nutritional, and inflammatory biomarkers; and third, to critically explore sources of heterogeneity among studies. A prespecified and central focus of this review is to rigorously test the hypothesis that the phenolic content of extra virgin olive oil serves as a significant modifier of the diet’s anti-inflammatory and potentially renoprotective effects. This work aims consolidate the current (often preliminary) evidence, highlight consistent patterns and unresolved discrepancies, and identify specific methodological and substantive gaps for future research.

## Method

### Study registration

This review was registered with PROSPERO (CRD420251124826) and reported in accordance with PRISMA guidelines.

### Data sources and searches

We systematically searched PubMed, Embase, Web of Science, Scopus, the Cochrane Central Register of Controlled Trials (CENTRAL), and ClinicalTrials.gov from inception to December 1, 2025, without language restrictions. The search strategy was designed to capture studies on the Mediterranean diet and chronic kidney disease, using a combination of controlled vocabulary and free-text keywords for the following core concepts: (1) Chronic kidney disease: (“chronic kidney disease” OR “chronic renal disease” OR CKD). (2) Mediterranean diet: (“Mediterranean diet” OR “Mediterranean dietary pattern”). (3) Study designs: (randomized controlled trial OR cohort OR prospective OR observational).

No exclusion terms (dialysis and transplant) were applied at the search stage to maximize sensitivity. The complete search strategy for each database is provided in Supplementary material S1. We also manually searched the reference lists of relevant articles.

### PICO framework

The systematic review was guided by the following PICO framework:

Population (P): Adults (≥18 years) with non-dialysis chronic kidney disease (CKD stages 1–5).
Intervention (I): A dietary intervention explicitly described and structured as a Mediterranean diet (MedDiet).
Comparison (C): A control diet (e.g., usual care, conventional renal diet, low-fat diet).
Outcomes (O): Primary: kidney function (eGFR, serum creatinine) and safety markers (potassium, phosphorus). Secondary: cardiometabolic, nutritional, and inflammatory biomarkers.

### Eligibility criteria

To ensure consistency in the included interventions, we applied an operational definition of the “Mediterranean diet intervention” based on its stated principles and core food components. Eligible studies were required to meet all of the following criteria:

1. Explicit Description: The intervention must be explicitly described in the source publication as a “Mediterranean diet” or a recognized derivative primarily based on its principles.
2. Adherence to Core Dietary Principles: The intervention design must reflect key characteristics of the traditional Mediterranean diet, primarily emphasizing plant-based foods and including at least two of the following core components: (1) Use of EVOO as the principal source of dietary fat. (2) High consumption of vegetables, fruits, legumes, and whole grains. (3) Moderate intake of fish and nuts. (4) Limited intake of red meat and processed foods.
3. Structured Delivery: The intervention must be delivered through at least one of the following structured modalities: provision of quantitative dietary prescriptions, personalized dietary counseling and education, or provision of meals or key food components adhering to the diet’s principles.

Furthermore, aligned with the exploratory aim of this meta-analysis, a prespecified subgroup analysis was conducted based on the phenolic content of the EVOO used. Pre-specified, hypothesis-driven subgroup analyses were conducted to investigate the proposed effect modification by EVOO phenolic content. Studies that specified the use of “high-phenolic” or “high minor polar compound” EVOO, or reported a total phenolic content above a common threshold (typically >500 mg of hydroxytyrosol derivatives per kg of oil), were categorized into the “high-phenolic EVOO” subgroup. Studies using EVOO without such specification or using common olive oil were categorized into the “unspecified/regular EVOO” subgroup.

The specific implementation of the interventions across included studies was heterogeneous, ranging from comprehensive dietary education to targeted food provision; key characteristics are detailed in Supplementary Table S1.

We included randomized controlled trials (RCTs), prospective cohort, or observational studies enrolling adults (≥18 years) with CKD stages 1–5 (KDIGO criteria) that compared a Mediterranean diet intervention to a control diet and reported at least one pre-specified renal, metabolic, or inflammatory outcome. We excluded studies involving dialysis or transplant patients, non-human studies, and publications without original data.

### Note on the specification and verification of EVOO phenolic content

The classification of ‘high-phenolic EVOO’ was operationally defined as described above. However, it is critical to acknowledge the heterogeneity in how this component was specified and verified across the included studies. Among the 10 included studies, only two (15, 16) provided detailed laboratory chromatography profiles confirming the phenolic content of the specific EVOO used exceeded the 500 mg/kg threshold. One study (17) provided analytical data showing a low phenolic content. The remaining seven studies either did not specify the phenolic content, relied on general ‘high-phenolic’ labels without verification, or did not have EVOO as a defined intervention component. Furthermore, factors affecting phenolic stability (storage time, conditions) were rarely reported. This variability means our ‘high-phenolic EVOO’ subgroup analysis is primarily driven by a small subset of methodologically specific studies, and the actual bioactive dose delivered in other trials is uncertain.

### Outcomes of interest

Primary outcomes were kidney function (eGFR, serum creatinine) and safety (serum potassium, phosphorus). Secondary outcomes included blood urea nitrogen (BUN), lipid profile, fasting glucose, blood pressure, body composition (weight, BMI, fat mass), and markers of nutrition and inflammation (serum albumin, C-reactive protein [CRP], hemoglobin).

### Data collection and risk of bias assessment

Continuous variable data (mean ± standard deviation or median with range) extracted from the included original studies are recorded in Supplementary Table S2. We noted that different studies reported biochemical indicators using varying units of measurement. The originally reported values and units were retained during data extraction. A row containing conversion formulas for common biochemical units is provided at the bottom of Supplementary Table S1 to facilitate interpretation. Two reviewers independently extracted data using standardized forms. Risk of bias was assessed using Cochrane RoB 2.0 for RCTs and ROBINS-I for non-randomized studies; disagreements were resolved by consensus or a third reviewer.

### Data analysis

To integrate studies with differently reported outcomes, we synthesized data using the mean difference (MD) in change from baseline as the unified effect measure, under a random-effects model (DerSimonian and Laird) (18). Heterogeneity was quantified with the I^2^ statistic (19). For studies lacking variance data for change scores, we imputed the variance assuming a correlation coefficient (r) of 0.7 between baseline and follow-up measurements, based on the moderate-to-high stability of renal and cardiometabolic markers in chronic conditions. To evaluate the robustness of this assumption, sensitivity analyses were performed using r = 0.4 and r = 0.9. Pre-specified subgroup analyses were conducted according to the phenolic content of EVOO used in the interventions. We planned to assess publication bias using funnel plots and Egger’s test if ≥10 studies were available for an outcome. Detailed formulas and analytical steps are provided in Supplementary material S2. For all continuous outcomes, the effect size is presented as the mean difference (MD) with its 95% confidence interval (CI). All biochemical data extracted from the original studies were converted to standard international (SI) units using established conversion formulas prior to analysis to ensure consistency in the pooled results.

## Result

### Study selection and characteristics

**Figure 1:**
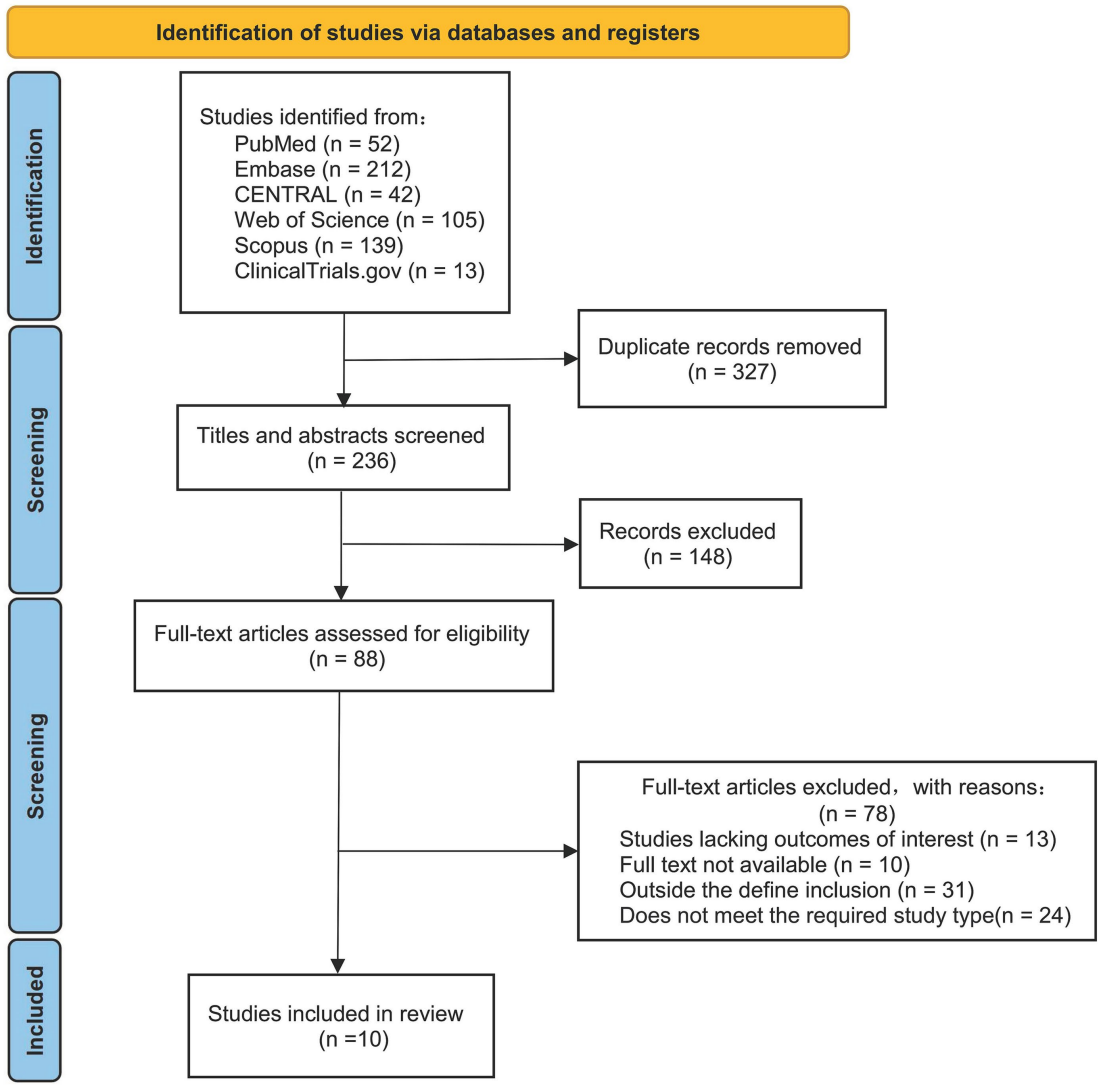
PRISMA flowchart.

The literature search yielded 563 records. After screening, 10 studies (6 RCTs, 4 observational studies, shown in Figure 1) involving 1,073 participants met inclusion criteria (15–17, 20–26). Study durations ranged from 8 weeks to 5 years. Participants had a mean age of 54–71 years, primarily CKD stages 3–5, with high prevalences of hypertension and type 2 diabetes. Most interventions (7/10) emphasized EVOO intake. Control groups received conventional CKD dietary advice or low-fat diets. Key study characteristics are summarized in Supplementary Table S1.

### Risk of bias and study quality assessment

The Cochrane Risk of bias tool (RoB 2.0) and the ROBINS-I tool were used to appraise the methodological quality of the randomized and non-randomized studies, respectively. Among the six RCTs, two were judged to be at high risk of bias, three raised some concerns, and one was at low risk. Key concerns pertained to deviations from intended interventions, primarily due to the infeasibility of blinding in dietary trials. All four non-randomized studies were at moderate to high risk of bias, with bias due to confounding being the predominant limitation. The absence of blinding in several studies also introduced potential bias in outcome measurement. A summary of the risk of bias assessment is provided in Supplementary Table S3.

### Synthesis of results

#### Effect on renal function and safety markers

**Figure 2:**
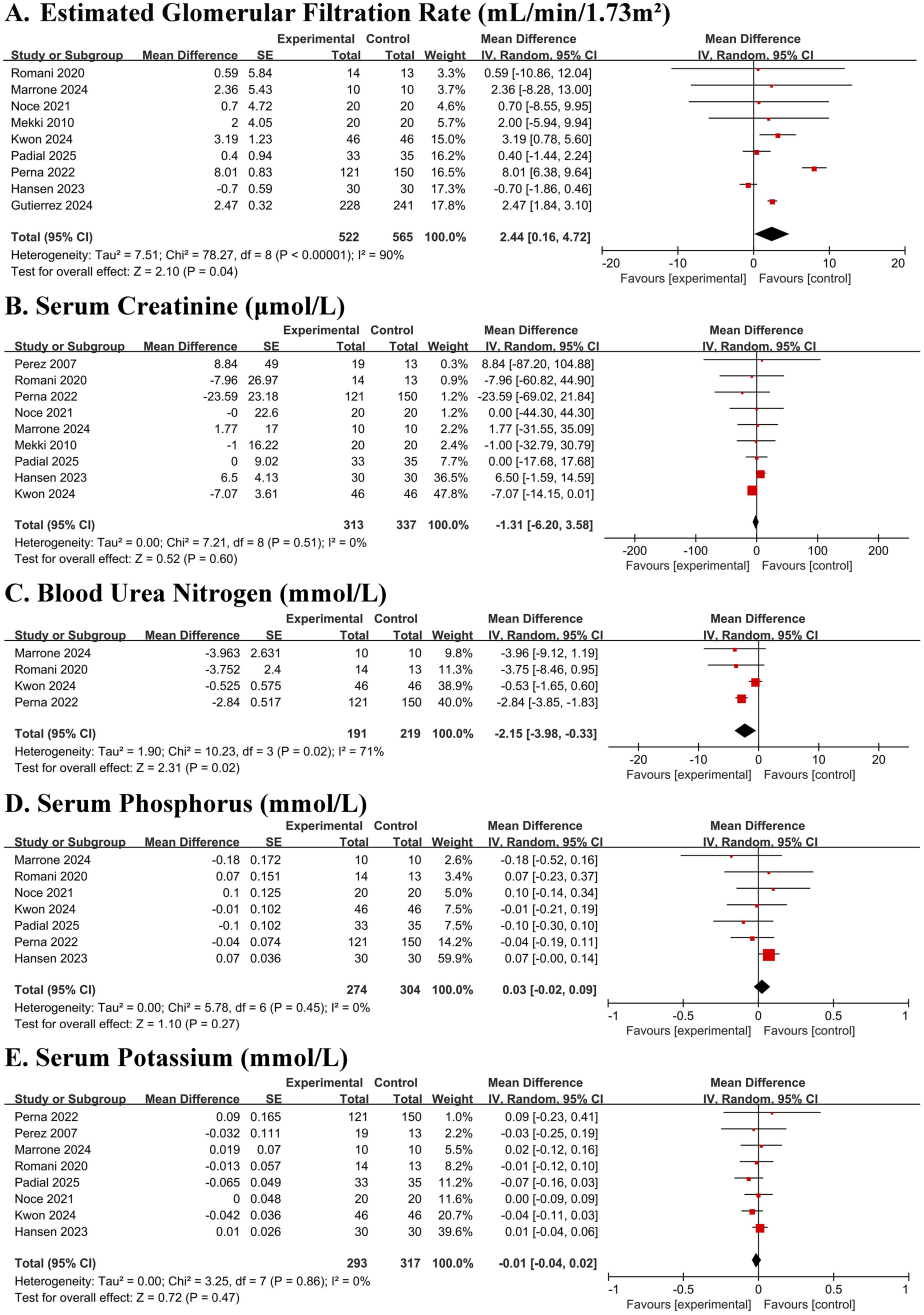
Forest plots of the effect of Mediterranean diet on renal function and safety markers in patients with chronic kidney disease. **(A)** Estimated glomerular filtration rate (eGFR). **(B)** Serum creatinine. **(C)** Blood urea nitrogen (BUN). **(D)** Serum phosphorus. **(E)** Serum potassium. Data are presented as mean difference with 95% confidence intervals (CIs). Squares represent individual study estimates; horizontal lines represent 95% CIs. The diamond represents the pooled random-effects estimate. The vertical dashed line indicates the line of no effect (mean difference = 0).

Pooled analysis demonstrated that adherence to a Mediterranean diet was associated with a statistically significant, albeit modest, improvement in estimated glomerular filtration rate (eGFR) (MD 2.44 mL/min/1.73 m^2^, 95% CI 0.16 to 4.72; *p* = 0.04) (Figure 2A). However, this finding was accompanied by considerable heterogeneity (I^2^ = 90%). A significant reduction in blood urea nitrogen (BUN) was also observed (MD − 2.15 mmol/L, 95% CI − 3.98 to −0.33; I^2^ = 71%; *p* = 0.02) (Figure 2C). No significant effects were found on serum creatinine, potassium, or phosphorus levels (all *p* > 0.05) (Figures 2B,D,E).

#### Effect on lipid profiles

**Figure 3:**
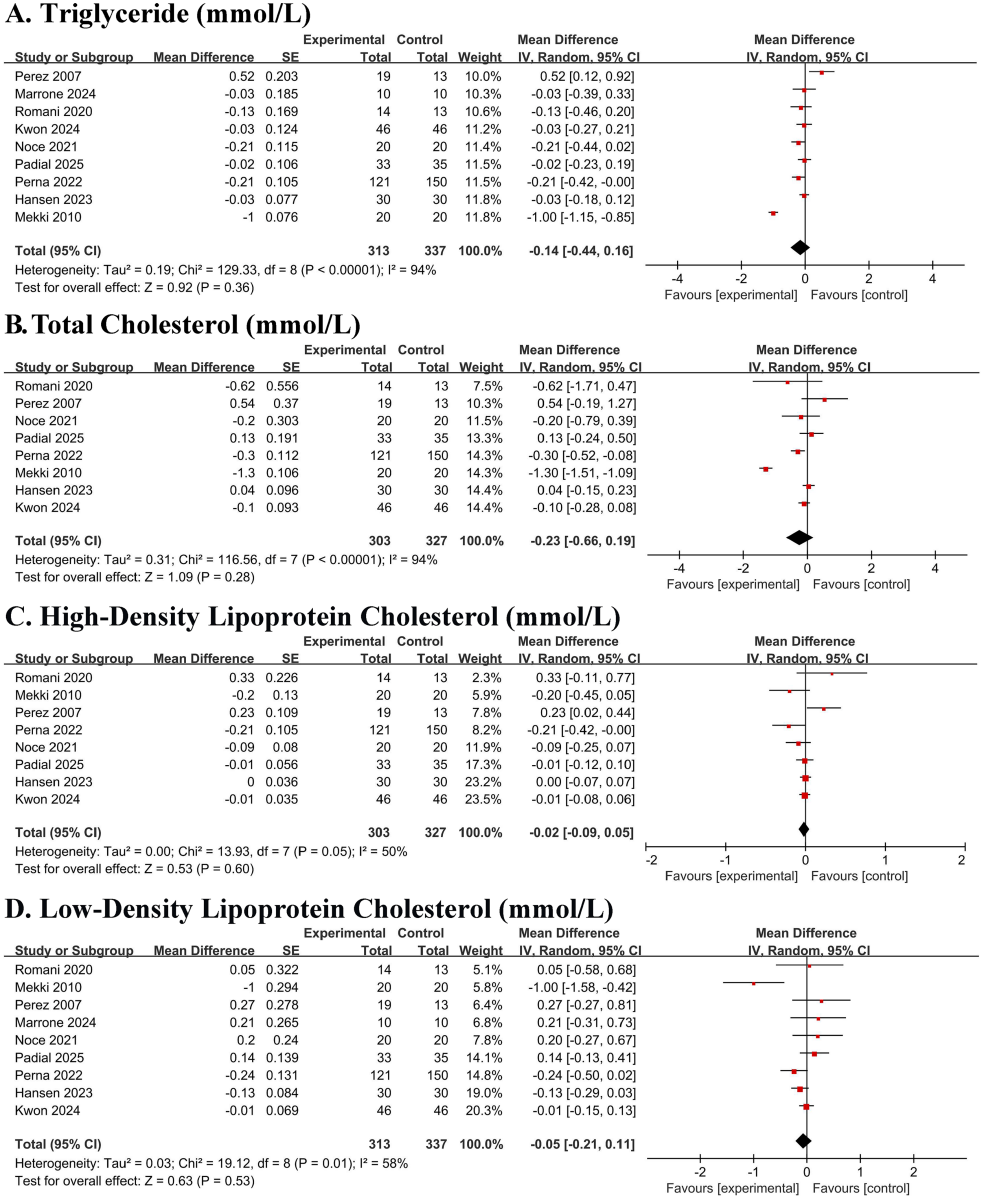
Forest plots of the effect of Mediterranean diet on lipid profiles in patients with chronic kidney disease. **(A)** Triglyceride. **(B)** Total cholesterol. **(C)** High-density lipoprotein cholesterol (HDL-C). **(D)** Low-density lipoprotein cholesterol (LDL-C). Data are presented as mean difference in mmol/L with 95% confidence intervals (CIs). Squares represent individual study estimates; horizontal lines represent 95% CIs. The diamond represents the pooled random-effects estimate. The vertical dashed line indicates the line of no effect (mean difference = 0).

The meta-analysis revealed no significant effects on triglycerides (MD − 0.14 mmol/L, 95% CI − 0.43 to 0.15; *p* = 0.36), total cholesterol (MD − 0.23 mmol/L, 95% CI − 0.66 to 0.19; *p* = 0.28), HDL-C (MD –0.01 mmol/L, 95% CI − 0.06 to 0.03; *p* = 0.60), or LDL-C (MD − 0.05 mmol/L, 95% CI − 0.22 to 0.11; *p* = 0.53), with considerable heterogeneity noted for lipid outcomes (I^2^ 58–94%) (Figures 3A–D).

#### Effect on cardiometabolic risk factors and body composition

**Figure 4:**
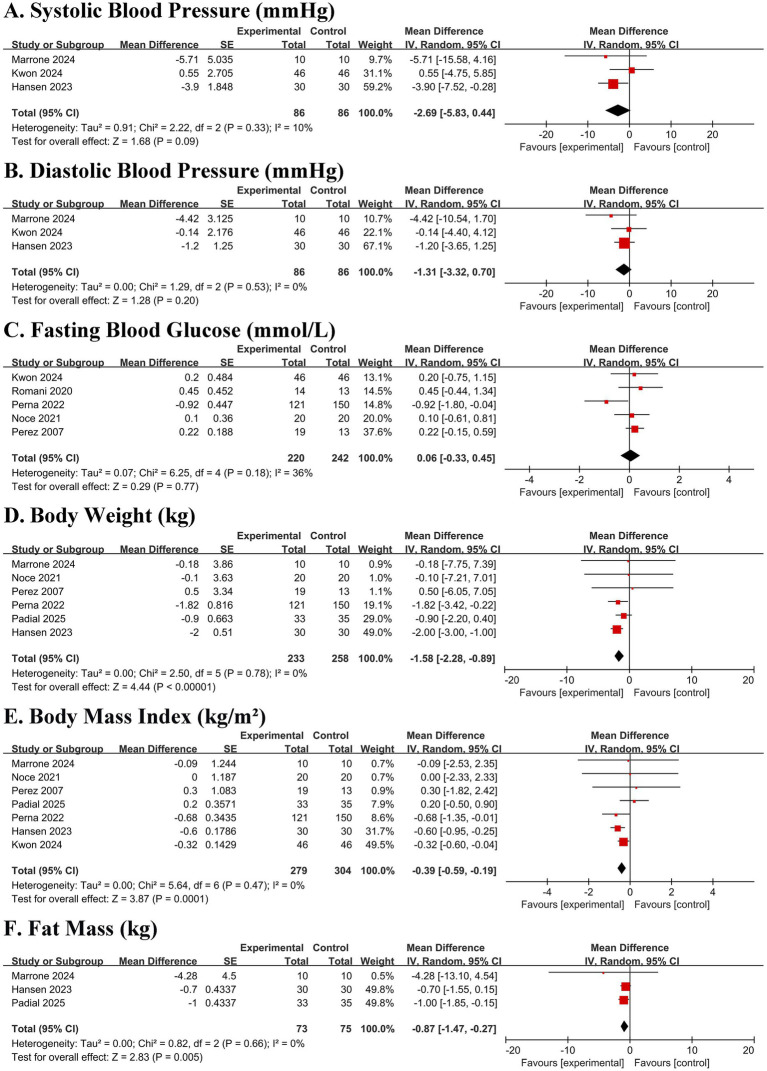
Forest plots of the effect of Mediterranean diet on cardiometabolic risk factors and body composition in patients with chronic kidney disease. **(A)** Systolic blood pressure (SBP). **(B)** Diastolic blood pressure (DBP). **(C)** Fasting blood glucose (FBG). **(D)** Body weight. **(E)** Body mass index (BMI). **(F)** Fat mass. Data are presented as mean difference with 95% confidence intervals (CIs). Squares represent individual study estimates; horizontal lines represent 95% CIs. The diamond represents the pooled random-effects estimate. The vertical dashed line indicates the line of no effect (mean difference = 0). The direction of effect for blood pressure is indicated (Mediterranean diet vs. control).

No significant effects were observed on systolic blood pressure (MD − 2.69 mmHg, 95% CI − 5.83 to 0.44; *p* = 0.09), diastolic blood pressure (MD − 1.31 mmHg, 95% CI − 3.32 to 0.70; *p* = 0.20), or fasting glucose (MD 0.06 mmol/L, 95% CI − 0.33 to 0.45; *p* = 0.77) (Figures 4A–C). In contrast, significant and consistent reductions were observed in body weight (MD − 1.58 kg, 95% CI − 2.28 to −0.89; *p* < 0.00001), BMI (MD − 0.39 kg/m^2^, 95% CI − 0.59 to −0.19; *p* = 0.0001), and fat mass (MD − 0.87 kg, 95% CI − 1.47 to −0.27; *p* = 0.005), with minimal heterogeneity (I^2^ = 0% for all) (Figures 4D–F).

#### Effect on nutritional and inflammatory markers

**Figure 5:**
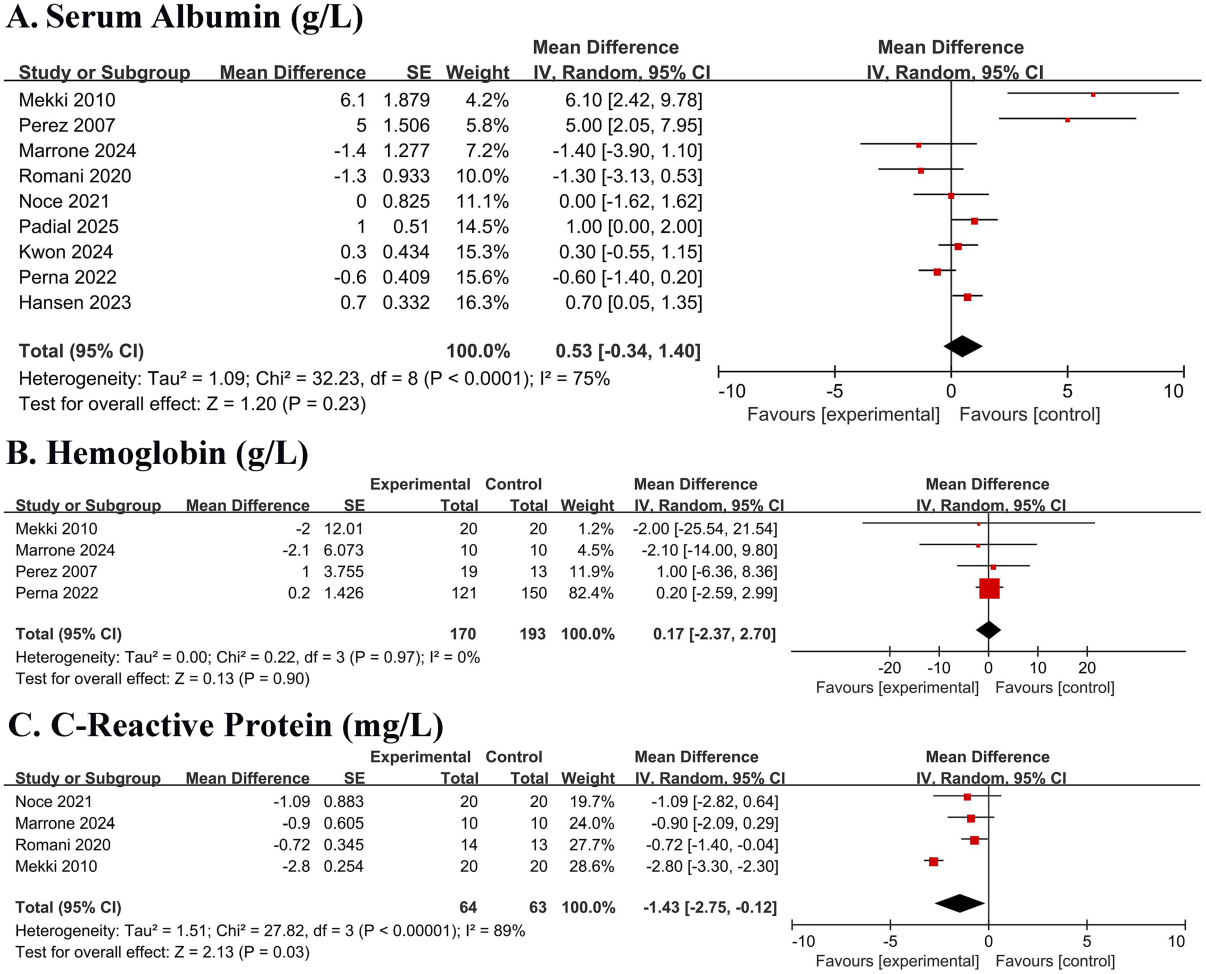
Forest plots of the effect of Mediterranean diet on nutritional and inflammatory markers in patients with chronic kidney disease. **(A)** Serum albumin. **(B)** Hemoglobin. **(C)** C-reactive protein (CRP). Data are presented as mean difference with 95% confidence intervals (CIs). Squares represent individual study estimates; horizontal lines represent 95% CIs. The diamond represents the pooled random-effects estimate. The vertical dashed line indicates the line of no effect (mean difference = 0).

No significant overall effect was detected for serum albumin (MD 0.53 g/L, 95% CI − 0.34 to 1.40; *p* = 0.23) or hemoglobin (MD 0.17 g/L, 95% CI − 2.37 to 2.70; *p* = 0.90) (Figures 5A,B). However, a significant reduction in C-reactive protein (CRP) was observed (MD − 1.43 mg/L, 95% CI − 2.75 to −0.12; *p* = 0.03), although with high heterogeneity (I^2^ = 89%) (Figure 5C).

#### Sensitivity and prespecified subgroup analyses

**Figure 6:**
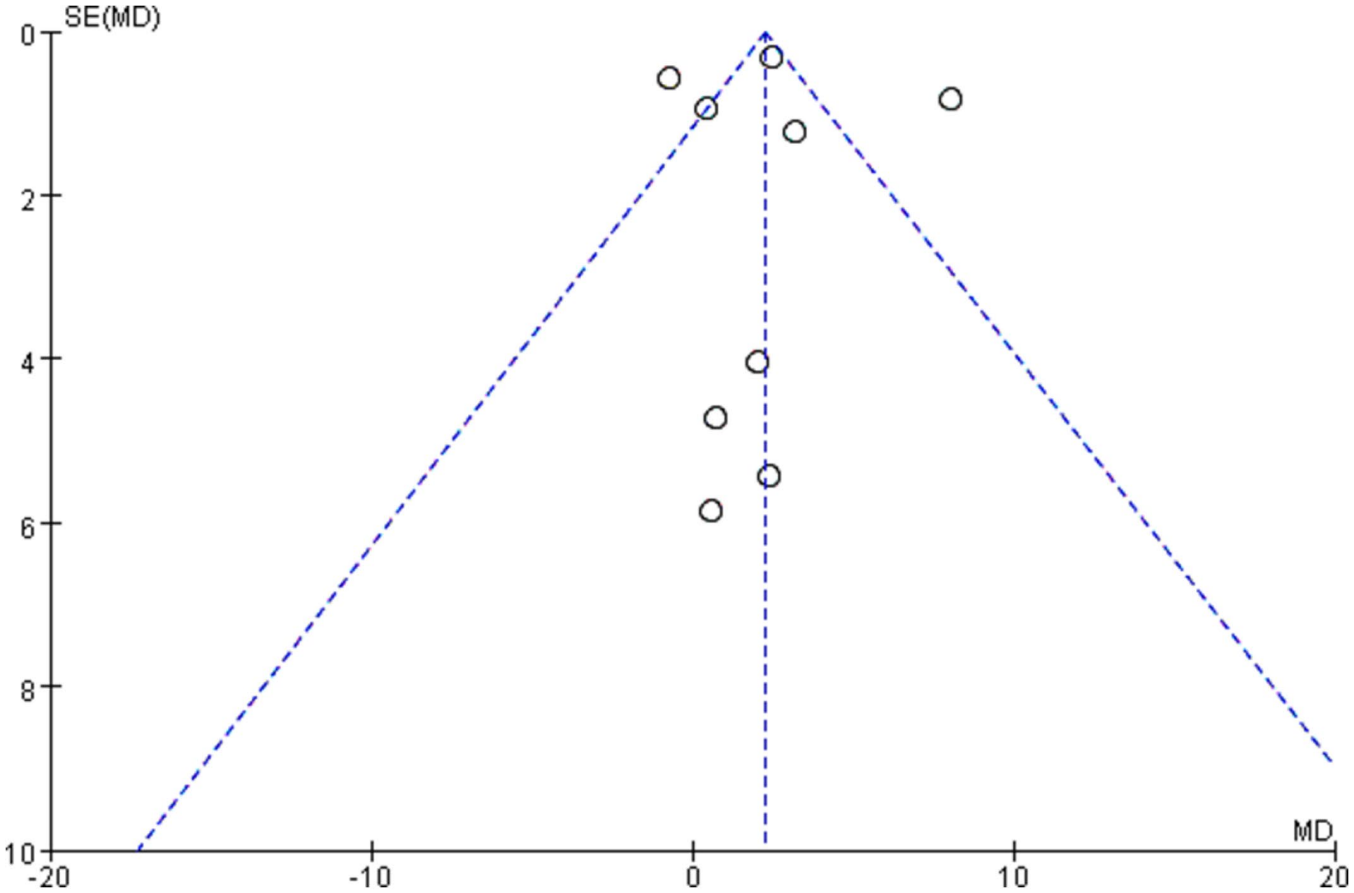
Funnel plot for visual assessment of potential publication bias (primary outcome: eGFR). The plot was constructed under the fixed-effect assumption. Each point represents an individual study’s effect estimate (mean difference in eGFR) plotted against its standard error. The solid vertical line represents the fixed-effect summary estimate, and the dashed oblique lines outline the expected 95% confidence interval for studies of a given standard error under the assumption of no heterogeneity. Visual inspection does not suggest substantial asymmetry. Statistical power to detect asymmetry is limited with fewer than 10 studies.

Sensitivity analyses confirmed the robustness of the findings regarding BUN and safety outcomes. For eGFR, leave-one-out analysis indicated that the significant pooled estimate was sensitive to the inclusion of a single large cohort study (21), underscoring the fragility of this finding in the context of high heterogeneity. To further evaluate the robustness of the primary renal outcome and address the influence of study design, a post-hoc sensitivity analysis was conducted by including only randomized controlled trials (RCTs) for eGFR. This analysis yielded a non-significant pooled estimate (MD 1.33 mL/min/1.73 m^2^, 95% CI − 0.44 to 3.09 *p* = 0.14), indicating that the statistically significant benefit observed in the main analysis is sensitive to the inclusion of observational data. Potential publication bias for the primary outcome (eGFR) was visually assessed using a funnel plot constructed under the fixed-effect assumption (Figure 6).

**Figure 7:**
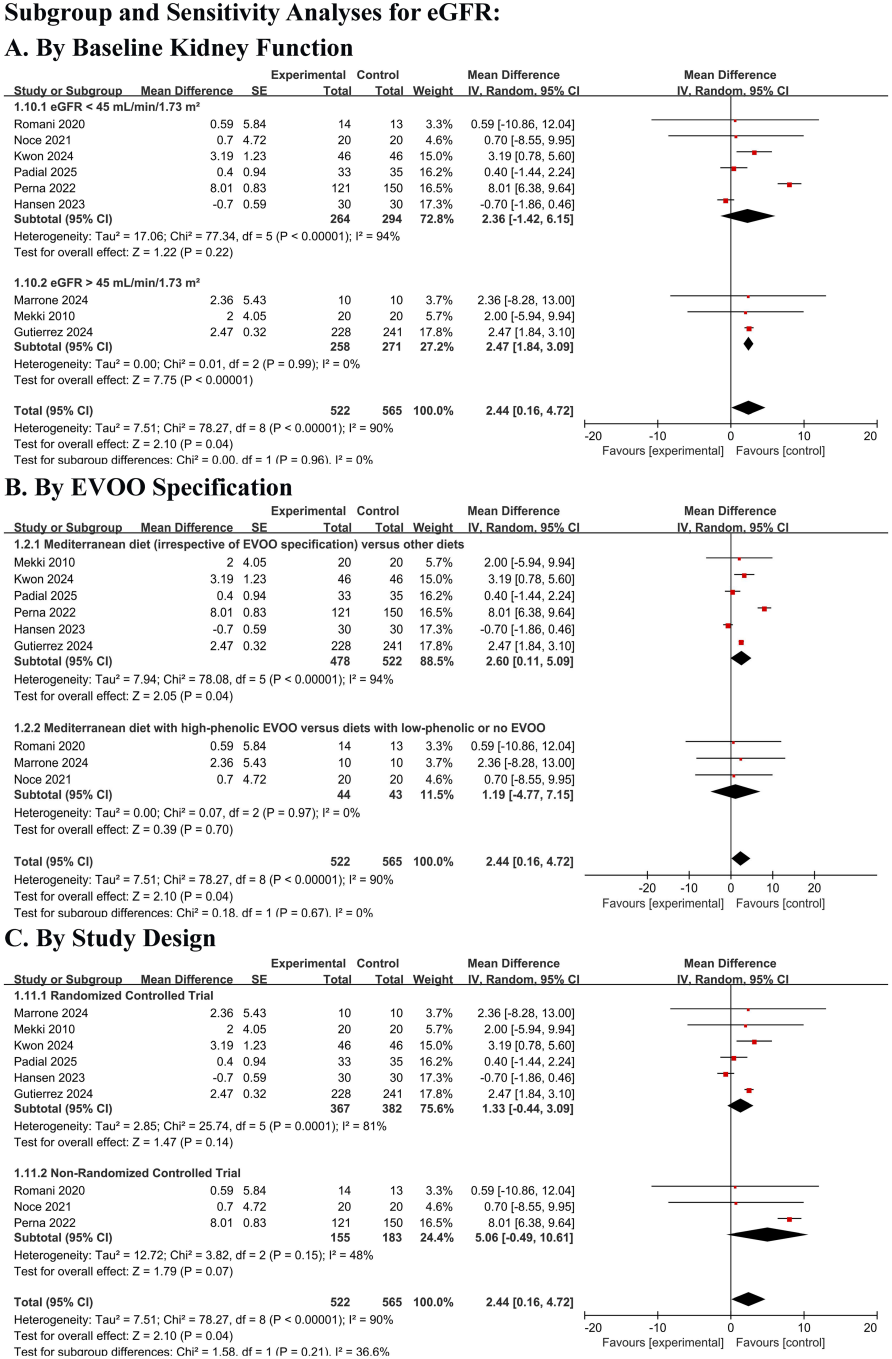
Subgroup and sensitivity analyses for the effect of Mediterranean diet on estimated glomerular filtration rate (eGFR). **(A)** Subgroup analysis by baseline kidney function. Patients were stratified into those with eGFR <45 mL/min/1.73 m^2^ (advanced CKD) and ≥45 mL/min/1.73 m^2^ (mild-to-moderate CKD). **(B)** Subgroup analysis by extra virgin olive oil (EVOO) specification. Interventions were categorized as “Mediterranean diet (irrespective of EVOO specification)” or “Mediterranean diet with high-phenolic EVOO”. **(C)** Subgroup analysis by study design. Studies were stratified as randomized controlled trials (RCTs) or non-randomized studies. Data are presented as mean difference in ml/min/1.73 m^2^ with 95% confidence intervals (CIs). Squares represent individual study or subgroup estimates; horizontal lines represent 95% CIs. The diamond represents the pooled random-effects estimate for each subgroup or the overall effect. The vertical dashed line indicates the line of no effect (mean difference = 0).

Pre-specified subgroup analyses for eGFR revealed a critical distinction (Figure 7). Stratification by baseline kidney function showed that the modest eGFR improvement was more consistent in patients with mild-to-moderate CKD (eGFR ≥45 mL/min/1.73 m^2^), whereas evidence in advanced CKD (eGFR <45 mL/min/1.73 m^2^) was weak and inconsistent. Subgroup analysis by study design indicated that effect estimates were larger in observational studies than in RCTs, a pattern consistent with potential residual confounding.

**Figure 8:**
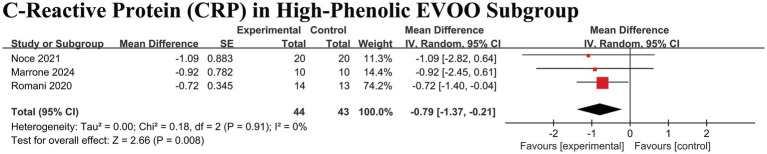
Forest plot of the effect of Mediterranean diet with high-phenolic extra virgin olive oil (EVOO) on C-reactive protein (CRP) in patients with chronic kidney disease. This analysis includes only interventions that explicitly used high-phenolic EVOO. Data are presented as mean difference in mg/L with 95% confidence intervals (CIs). Squares represent individual study estimates; horizontal lines represent 95% CIs. The diamond represents the pooled random-effects estimate. The vertical dashed line indicates the line of no effect (mean difference = 0).

In striking contrast, the anti-inflammatory effect (reduction in CRP) demonstrated clear component-specificity. When the analysis was restricted to interventions that explicitly included high-phenolic extra virgin olive oil (EVOO), a precise and significant reduction in CRP was observed (MD − 0.79 mg/L, 95% CI − 1.37 to −0.21; *p* = 0.008; I^2^ = 0%) (Figure 8).

**Figure 9:**
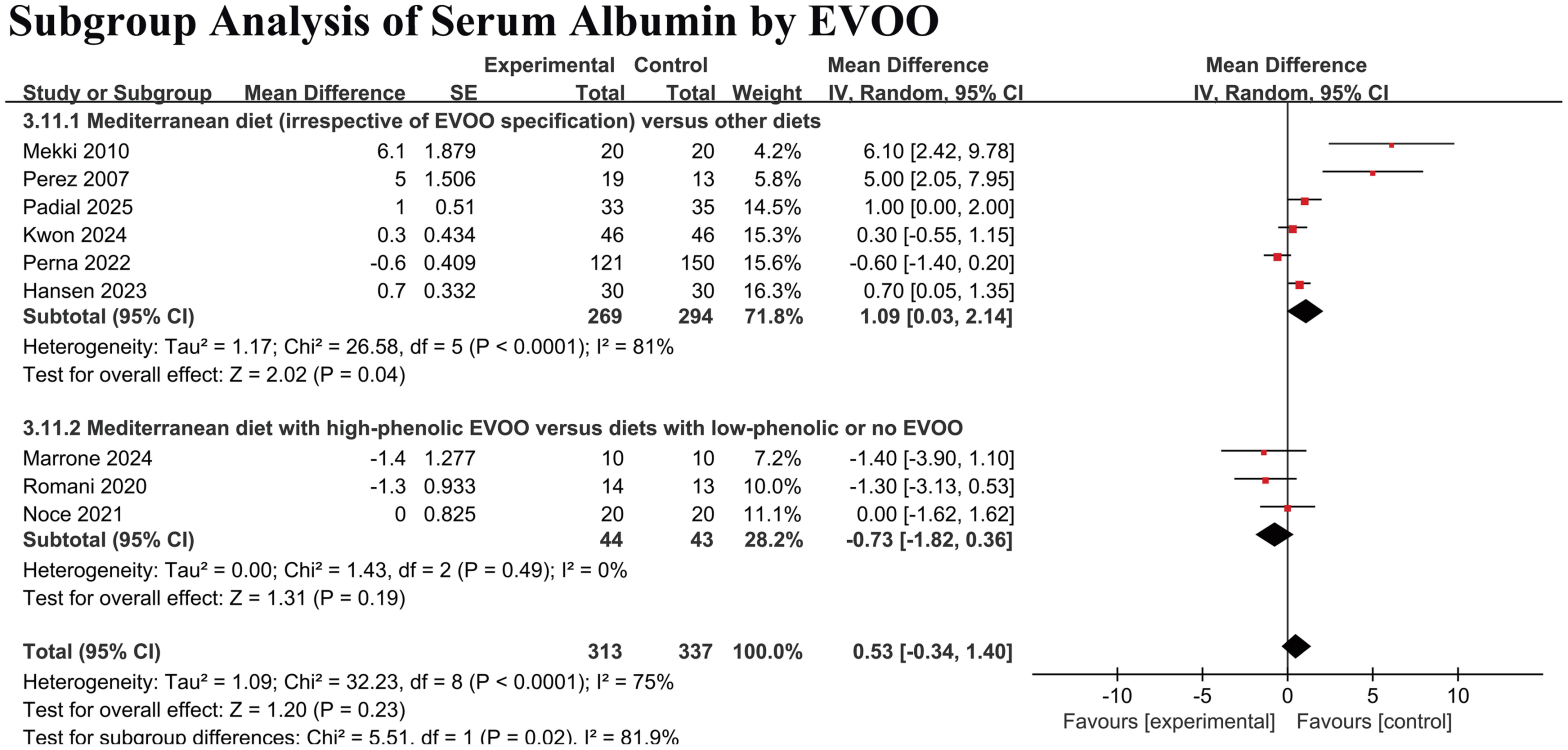
Subgroup analysis of the effect of Mediterranean diet on serum albumin, stratified by the specification of extra virgin olive oil (EVOO). Interventions were categorized as “Mediterranean diet (irrespective of EVOO specification)” or “Mediterranean diet with high-phenolic EVOO”. Data are presented as mean difference in g/L with 95% confidence intervals (CIs). Squares represent individual study estimates; horizontal lines represent 95% CIs. The diamond represents the pooled random-effects estimate for each subgroup. The vertical dashed line indicates the line of no effect (mean difference = 0). A test for subgroup differences indicated a statistically significant difference between the two strata (*p* = 0.02).

Conversely, for serum albumin, subgroup analysis indicated a benefit in the broader diet subgroup but not in the high-phenolic EVOO subgroup, with a statistically significant interaction between subgroups (P for subgroup difference = 0.02) (Figure 9).

## Discussion

This meta-analysis quantifies the effects of the Mediterranean diet in adults with nondialysis CKD. The principal findings are a modest but potentially clinically meaningful improvement in eGFR, significant benefits on body composition, a specific reduction in systemic inflammation linked to high-phenolic EVOO, and a reassuring safety profile regarding serum potassium and phosphorus. Notably, improvements in traditional cardiometabolic surrogates, such as lipids and blood pressure, were not observed in this population.

The observed eGFR benefit (MD 2.44 mL/min/1.73 m^2^), while modest, is clinically relevant. In the context of CKD, an annual eGFR decline exceeding 2–4 mL/min/1.73 m^2^ is clinically defined as rapid progression (27, 28). The effect size observed in our study approximates this threshold, which is considered clinically significant in slowing disease progression (29). This suggests that adoption of a Mediterranean dietary pattern could meaningfully attenuate the rate of kidney function loss over time, representing a non-pharmacological strategy with the potential to modify disease trajectory. This finding extends prior observational data by providing a quantitative estimate from intervention studies, reinforcing the diet’s potential role in decelerating CKD progression.

The divergent effects, namely benefits on body composition and inflammation but null effects on lipids and blood pressure, require a multifactorial explanation. First, the “active comparator” issue is paramount. In landmark trials such as PREDIMED (7), control groups received minimal dietary advice. In contrast, control groups in the included CKD trials typically received structured, renal-specific dietary counseling aimed at mitigating cardiovascular risk, which may narrow the expected effect size on these metabolic parameters (30). Second, methodological limitations prevalent in nutritional trials, including imperfect adherence, potential contamination between groups, and non-blinded outcome assessment, can systematically bias results toward the null. Third, the high background use of potent cardiometabolic medications in this advanced CKD population likely exerts a ceiling effect, potentially obscuring further modest improvements from dietary modification (31, 32). Therefore, the negative findings for lipids and blood pressure should be interpreted cautiously and may reflect trial context rather than a true absence of biological effect.

Furthermore, our prespecified subgroup analysis revealed a critical nuance: the modest eGFR benefit was not statistically significant in patients with advanced CKD, defined as an eGFR <45 mL/min/1.73 m^2^. This stage-specific discrepancy warrants explanation. In advanced CKD, pathophysiology is dominated by factors such as established metabolic acidosis, severe mineral and bone disorder, and the accumulation of protein-bound uremic toxins. While the MedDiet’s components, particularly its high fiber content and the shift toward plant-based proteins with lower phosphorus bioavailability, are theoretically advantageous for mitigating these issues, the magnitude of these derangements in late-stage disease may exceed the corrective capacity of dietary intervention alone within the timeframe of most trials. Additionally, the high prevalence of comorbidities and polypharmacy, including potent renin-angiotensin-aldosterone system inhibitors and diuretics, creates a high therapeutic ceiling, making additional incremental benefits from diet more difficult to detect. Future studies should investigate whether the MedDiet, particularly when initiated earlier or integrated as a core component of multimodal therapy, can modify the trajectory of advanced CKD.

Our exploratory analyses provide compelling evidence for a specific anti-inflammatory effect of high-phenolic EVOO, evidenced by a homogeneous and significant reduction in CRP in the relevant subgroup. This aligns with the established pharmacology of EVOO polyphenols, which have been shown to inhibit key pro-inflammatory pathways such as NF-κB and NLRP3 inflammasome activation, and to reduce oxidative stress (9, 33–37). The anti-inflammatory properties of high-phenolic EVOO are increasingly recognized as a key mediator of its cardioprotective effects. This finding suggests that for CKD patients, a condition fueled by chronic inflammation (38), the inclusion of high-phenolic EVOO is a critical component for realizing this specific benefit. Conversely, the improvements in eGFR and serum albumin appeared more closely associated with the broader dietary pattern than with high-phenolic EVOO specifically. This suggests benefits on renal function and nutritional status may arise from the synergy of multiple factors inherent to the diet: a favorable electrolyte and acid–base profile, increased fiber intake, and a shift toward plant-based proteins (39–44). Specifically: (1) the higher fiber content from fruits, vegetables, and legumes may improve the gut microbiome, increase the production of short-chain fatty acids with anti-inflammatory properties, and beneficially modulate the metabolism of uremic toxins (45, 46); (2) plant-based proteins, compared to animal proteins, generate a lower dietary acid load and provide phosphorus in a less bioavailable form, which can help mitigate metabolic acidosis and hyperphosphatemia, both key drivers of CKD progression and mineral bone disease (40, 41, 47). This exemplifies the concept of food synergy (48), whereby the combined effects of the diet’s components, operating through complementary pathways such as gut microbiota modulation, acid base regulation, and anti-inflammatory action, may be greater than the sum of their individual effects, collectively contributing to renal protection.

An equally important finding is the lack of significant adverse effect on serum potassium or phosphorus, alleviating a common concern regarding plant-rich diets in CKD. The high fiber content and lower bioavailability of minerals from plant sources likely explain this safety profile(40–42, 44).

### Limitations

Our findings must be interpreted in light of several important limitations. First, the evidence base remains limited by a small number of randomized controlled trials (RCTs), and a substantial proportion of included studies exhibited moderate-to-high risk of bias, particularly due to challenges in blinding and residual confounding. This highlights an urgent need for larger, rigorously designed RCTs with longer follow-up durations. Second, the observed high statistical heterogeneity for several outcomes complicates the interpretation of pooled estimates. While our prespecified subgroup analyses offered plausible explanations, residual heterogeneity suggests unmeasured effect modifiers: a key priority for future individual participant data meta-analyses. Third, the phenolic content of EVOO was often poorly reported and variable across studies, limiting the precision of our component-specific conclusions and underscoring the necessity for standardized reporting of bioactive dietary components in nutritional trials. Fourth, the evidence synthesized herein is constrained by the nature of available primary studies. The interventions were predominantly short- to mid-term (median ≤6 months), which is sufficient to detect changes in body composition but likely insufficient for full metabolic and hemodynamic adaptations to manifest, potentially explaining the disconnect between weight loss and null effects on blood pressure and lipids. More fundamentally, large-scale, long-term randomized controlled trials (RCTs) specifically designed for the CKD population are absent. While landmark RCTs like PREDIMED have established the cardiovascular benefits of the MedDiet in broader at-risk populations, the direct translation and durability of these effects in the distinct pathophysiology of advanced CKD remain to be conclusively established. Our findings, therefore, highlight a critical evidence gap and underscore the need for definitive trials in this high-risk group, rather than refuting the diet’s established benefits in general cardiometabolic health. Finally, we did not formally grade the certainty of evidence, which should be considered when interpreting the strength of our conclusions. Collectively, these limitations underscore the need for cautious interpretation of our findings and highlight specific avenues for improving the evidence base in future research.

### Clinical implications and future research

Our findings support the Mediterranean diet as a safe, palatable, and potentially renoprotective dietary pattern in CKD management. It should be framed positively as an evidence-based strategy for overall health rather than a restrictive “renal diet.” Clinicians may consider recommending the diet with an emphasis on incorporating high-phenolic EVOO to target chronic inflammation. Future research must address the current evidence gaps. Priority should be given to large, long-term, well-designed RCTs with hard clinical endpoints. These trials should:

1. Isolate the effect of EVOO phenolic content by employing a 2 × 2 factorial design that directly compares a Mediterranean diet with high-phenolic EVOO versus one with low-phenolic EVOO, and, if ethically feasible, versus a matched control diet without EVOO supplementation.

2. Explore therapeutic synergy by investigating potential additive or synergistic interactions between a Mediterranean diet (particularly with high-phenolic EVOO) and emerging nephroprotective pharmacotherapies, such as SGLT2 inhibitors and GLP-1 receptor agonists (49, 50).

3. Enhance real-world applicability by developing and testing a pragmatically adapted “CKD-tailored Mediterranean diet” that integrates renal-specific nutritional considerations within the dietary pattern. Such studies should employ innovative designs to optimize long-term adherence and assess effectiveness in diverse clinical settings.

## Data Availability

The original contributions presented in the study are included in the article/Supplementary material, further inquiries can be directed to the corresponding authors.
