# Supplementary material for: Mediterranean diet with high-phenolic EVOO slows kidney function decline and reduces inflammation in nondialysis CKD: a meta-analysis
Source: Front Nutr. 2026 Mar 2;13:1792390. doi: 10.3389/fnut.2026.1792390 (PMC12989350; doi:10.3389/fnut.2026.1792390)
Supplement: Supplementary file 1 [file Table_1.docx]

**Table S1. Key characteristics of the 10 included studies.**

| **No.** | **Study ID** | **Country** | **Study Type** | **Population** | **Exclusion criteria** | **Sample Size**  **(I/C)** | **Baseline eGFR**  **(mL/min/1.73㎡)** | **Intervention** | **Control** | **Key Dietary Prescription** | **Intervention Delivery&Adherence Assessment** | **KeyComorbidities** |
| --- | --- | --- | --- | --- | --- | --- | --- | --- | --- | --- | --- | --- |
| 17 | Pérez Bañasco et al.(2007) | Spain | Pilot RCT | CKD stage 4-5Year 60.8±12.8Male 50% | DM,immunodeficiencies,graft recipients,BMI>40,recent anti-inflammatory use. | 19/13 | 18.25±7.50 | Olive oil blend«oHo» | Usual diet(incl.habitual olive oil) | -Dose:60 mL/day«oHo»(3x20 mL,raw).-Diet:Usual diet,no other raw oils permitted.-Oil Composition:High MUFA(~78%oleic acid),rich in polyphenols(e.g.,hydroxytyrosol),phytosterols,vitamin E.) | -Delivery:Provided oil,syringe,consumption diary.-Assessment:Lab parameters(renal,lipid,glucose)at Day 0&30.30-day post-intervention follow-up.-Duration:30 days active+30 days follow-up. | Hypertension |
| 18 | Mekki et al.(2010) | Algeria | RCT | 60<eGFR<89Year 59.5±11.05Male 55% | Diabetic nephropathy,thyroid disease,anti-inflammatory/antioxidant use. | 20/20 | 75±15 | MD-adapted nutritional advice | Standard K/DOQI advice | -Goals:Energy~28.7 kcal/kg/d;Protein 0.75 g/kg/d.-MD Focus:↑MUFA/PUFA/fiber.Encourage EVOO,nuts,whole grains,fruits,vegetables,fish.-Education:Lists of high Na/K/P foods provided. | -Delivery:Personalized MD counseling by trained personnel.-Assessment:Nutritional surveys(recall/record)at baseline,30,60,90 days.-Duration:90 days. | Dyslipidemia 100% |
| 19 | Romani et al.(2020) | Italy | Non-randomized intervention | CKD stage 1-4Conservative therapyYear 68.4±11.9 | Active cancer,HIV/HBV/HCV,acute inflammation,BMI<18,pregnancy. | 14/13 | 36.2±18.6 | High-MPC EVOO(MOS:100%Moraiolo) | Lower-MPC EVOO(TB:Blend) | -Diet:Standard Italian MD with protein control per CKD stage.-Intervention:40 mL/day assigned EVOO as sole plant lipid.-MPC Content:MOS 706.83 mg/L;TB 496.80 mg/L(both>250 mg/kg hydroxytyrosol). | -Delivery:EVOO provided.-Assessment:PREDIMED&IPAQ questionnaires(baseline,9 wks);lab tests(renal,oxidative stress,lipids)at T0&T1.-Duration:9 weeks. | NR |
| 20 | Noce et al.(2021) | Italy | Pilot study | CKD stage 1-4Conservative therapyYear 69.6±11.9Male 62.5% | Active cancer,HIV/HBV/HCV,acute inflammation,BMI<18,pregnancy. | 20/20 | 36.1±18.5 | High-MPC EVOO(Synergy) | Medium-MPC EVOO(Luxolio) | -Dose:40 mL/day EVOO(raw).-Diet:Italian MD with protein control.-Fluid:Water intake 1000–1500 mL/day. | -Delivery:EVOO provided.-Assessment:PREDIMED,IPAQ,24-hr dietary recall at all timepoints.-Duration:9 weeks consumption+2-month washout. | NR |
| 21 | Perna et al.(2022) | Bahrain | Retrospectivecohort | Confirmed CKDYear 66.5±13.6Male 60.10% | Dropouts,single/transplanted kidney,AKI,stones,liver disease,PKD,cancer,age<18,missing data. | 121/150 | 31.21±14.99 | Personalized CKD-adapted MD | Generic dietary advice(nephrologist) | -Protein:≤0.8 g/kg/d(↑plant source).-Energy:30–35 kcal/kg/d.-Electrolytes:Na<2.3 g/d;P 0.8–1 g/d;K individualized.-Fat/Fiber:Fat<30%E(SFA<10%);Fiber 25–38 g/d. | -Delivery:Face-to-face counseling by renal dietitian;individualized care plan.-Assessment:Monthly follow-up via FFQ.-Duration:Mean 6 months(range 0–15). | HTN:87.8%DM:79.7%Heart dis.:19.3%Obesity(BMI>30) |
| 22 | Hansen et al.(2023) | Denmark | RCT | eGFR 20–45Year 54.5±12.5Male 48.5% | Phosphate binders,metabolic disorders,recent chemo,pregnancy,allergies,vegan diet. | 30/30 | 34.1±9.5 | New Nordic Renal Diet(NNRD:arefined extension of the MD) | Low-Fat Diet(LFD) | -Goals:Energy 30–35 kcal/kg/d;Protein 0.8 g/kg/d;Fat<35%E;NaCl<5 g/d;P~850 mg/d.-Composition:80%plant-based,no red meat,>95%organic,no additives. | -Delivery:Weekly home food/recipe delivery(5 days/wk);dietitian guidance.-Assessment:Monthly 48-hr dietary records(compliance score);24-hr urine(P,Na,urea).-Duration:26 weeks. | Metabolic syndromeI:53%,C:57% |
| 23 | Kwon et al.(2024) | SouthKorea | Pilot crossover RCT | CKD stage 3-4Year 66.1±14.6Male 56% | Kidney replacement therapy,K≥6.0 mEq/L,food allergies. | 46(crossover) | 39.35±13.31 | MEDI-POB Diet | Conventional Diet | -Protein:0.8 g/kg BW.-Na:≤2,000 mg/d.-Fat:High MUFA(EVOO),low SFA.-Fiber:High intake encouraged. | -Delivery:Home-delivered meals(5 days/wk);individualized education.-Assessment:24-hr recall;MD-adherence questionnaire;mobile app feedback.-Duration:4-wk intervention/arm,4-wk washout,12-wk total. | HTN:84–92%DM:64%Obesity(BMI 26–27) |
| 24 | Marrone et al.(2024) | Italy | Pilot RCT | CKD stage 2-4BMI 18.5-24.9Year 62.35±7.50Male 67.5% | Active cancer,HIV/HBV/HCV,refusal,acute inflammation,hyperkalemia. | 10/10 | 46.0±16.7 | Functional Food(FF)Bars | Usual Care | -Intervention:Two antioxidant-rich bars/day(EVOO,fruit,vegetables,grape pomace/seeds,olive leaf).-Diet:No specific macronutrient restrictions. | -Delivery:Daily bar consumption.-Assessment:Compliance via daily intake reporting.-Duration:12 weeks. | Arterial HTN,Dyslipidemia |
| 25 | Gutierrez-Mariscal et al.(2024) | Spain | RCT(single-blind) | eGFR≥30Year 61.2±0.3Male 80.93% | eGFR<30,extreme energy intake. | 228/241 | 86.5±0.7 | Mediterranean Diet(MedDiet) | Low-Fat Diet(LFD) | -MedDiet:Fat≥35%E(22%MUFA),Carbs≤50%,Protein 15%.Rich in EVOO,vegetables,fruits,legumes,nuts,fish.-LFD:Fat<30%E(12-14%MUFA),Carbs≥55%,Protein 15%.NCEP guidelines.-Both:Cholesterol<300 mg/d,no calorie restriction. | -Delivery:Intensive individual counseling by study dietitians.-Assessment:Repeated FFQ and 3-day food records.-Duration:5 years. | CAD,T2D(all),HTN,Obesity |
| 26 | Padial et al.(2025) | Spain | Pilot RCT | CKD stage 4-5eGFR<30Year 70.1(57.8,76.4)Male 68% | Active cancer,concurrent studies,pregnancy,recent hospitalization,mental illness,substance abuse. | 35/33 | 22.0(21.0–24.0) | Nutrition Education Program(coaching based) | General MD advice(single session) | -Diet:Modified MD for CKD 4-5.Protein 0.8 g/kg IBW/d.-Focus:Whole grains,vegetables,fruits,legumes,EVOO,nuts,fish.Avoid ultra-processed/high-P/K foods. | -Delivery:Initial counseling+3 workshops(month 1);weekly phone/text follow-up with coaching.-Assessment:PREDIMED score;labs;QoL(KDQOL-36),psychosocial(HADS,SEMCD,KKIS)questionnaires.-Duration:3 months. | DM:43%HTN:96% |
| Abbreviations:AKI,acute kidney injury;BMI,body mass index;BW,body weight;CAD,coronary artery disease;CKD,chronic kidney disease;DM,diabetes mellitus;eGFR,estimated glomerular filtration rate;EVOO,extra virgin olive oil;FFQ,food frequency questionnaire;HBV,hepatitis B virus;HCV,hepatitis C virus;HIV,human immunodeficiency virus;HTN,hypertension;I,intervention group;C,control group;IBW,ideal body weight;IPAQ,International Physical Activity Questionnaire;K,potassium;LFD,low-fat diet;MD,Mediterranean diet;MPC,minor polar compound;MUFA,monounsaturated fatty acid;Na,sodium;NCEP,National Cholesterol Education Program;NNRD,New Nordic Renal Diet;NR,not reported;P,phosphorus;PKD,polycystic kidney disease;PUFA,polyunsaturated fatty acid;QoL,quality of life;RCT,randomized controlled trial;SFA,saturated fatty acid;T2D,type 2 diabetes.Notes:a Data presented as mean±standard deviation or median(interquartile range)as reported in the original studies.Age and sex are summarized for the overall study population.CKD stage or eGFR range is per the original study definition.b NR,not reported.c Crossover trial:The sample size indicates the total number of participants who received both interventions sequentially. | | | | | | | | | | | | |
