# Supplementary material for: Mediterranean diet with high-phenolic EVOO slows kidney function decline and reduces inflammation in nondialysis CKD: a meta-analysis
Source: Front Nutr. 2026 Mar 2;13:1792390. doi: 10.3389/fnut.2026.1792390 (PMC12989350; doi:10.3389/fnut.2026.1792390)
Supplement: Supplementary file 2 [file Table_2.docx]

Search Strategy for Systematic Review: "Renal, Metabolic, and Anti-Inflammatory Effects of the Mediterranean Diet in Non-dialysis Chronic Kidney Disease"

Reporting Guideline: This search strategy is reported in accordance with the PRISMA-S extension.

**1. Overall Information**

Date of Search Execution: 2025.9.10

Databases Searched: PubMed, Web of Science (Core), Scopus, Cochrane Central Register of Controlled Trials (CENTRAL), Embase, ClinicalTrials.gov

Grey Literature: ClinicalTrials.gov was searched for ongoing or completed trials.

Other Sources: Reference lists of included studies and relevant review articles were manually screened.

Language Restrictions: None applied.

Search Filters: Human studies.

**2. Detailed Search Queries by Database**

**2.1. PubMed (via NCBI)**

Date Searched: 2025.9.10

Records Retrieved: 52

#1 "Renal Insufficiency, Chronic"[Mesh] OR "chronic kidney disease"[tiab] OR "chronic renal disease"[tiab] OR CKD[tiab] OR ESRD[tiab] OR ESKD[tiab]

#2 "Diet, Mediterranean"[Mesh] OR "Mediterranean diet"[tiab] OR "Mediterranean dietary pattern"[tiab]

#3 randomized controlled trial[pt] OR controlled clinical trial[pt] OR randomized[tiab] OR randomised[tiab] OR placebo[tiab] OR clinical trial[tiab] OR "clinical trials as topic"[mesh:noexp] OR trial[ti]

#4 observational study[pt] OR "observational studies as topic"[Mesh] OR cohort[tiab] OR prospective[tiab] OR longitudinal[tiab]

#5 #3 OR #4

#6 #1 AND #2 AND #5

#7 Filters: Humans

**2.2. Web of Science (Core)**

Date Searched: 2025.9.10

Records Retrieved: 105

Search Query:

(TS=("chronic kidney disease" OR "chronic renal disease" OR CKD OR ESRD OR ESKD OR "renal insufficiency"))

AND

(TS=("Mediterranean diet" OR "Mediterranean dietary pattern"))

AND

(TS=(random* OR RCT OR "clinical trial" OR trial OR cohort OR prospective OR observational OR longitudinal))

Indexes=SCI-EXPANDED, SSCI, A&HCI, CPCI-S, CPCI-SSH, ESCI Timespan=All years

**2.3. Scopus**

Date Searched: 2025.9.10

Records Retrieved: 139

Search Query:

( TITLE-ABS-KEY ( "chronic kidney disease" OR "chronic renal disease" OR ckd OR esrd OR eskd OR "renal insufficiency" ) )

AND

( TITLE-ABS-KEY ( "mediterranean diet" OR "mediterranean dietary pattern" ) )

AND

( TITLE-ABS-KEY ( random* OR rct OR "clinical trial" OR trial OR cohort OR prospective OR observational OR longitudinal ) )

**2.4. Cochrane Central Register of Controlled Trials (CENTRAL, via Cochrane Library)**

Date Searched: 2025.9.10

Records Retrieved: 42

Search Query:

#1 MeSH descriptor: [Renal Insufficiency, Chronic] explode all trees

#2 ("chronic kidney" OR "chronic renal" OR CKD OR ESRD OR ESKD):ti,ab,kw

#3 #1 OR #2

#4 MeSH descriptor: [Diet, Mediterranean] explode all trees

#5 ("Mediterranean Diet" OR "Mediterranean dietary pattern"):ti,ab,kw

#6 #4 OR #5

#7 #3 AND #6

Publication Year from 1900 to 2025， in Trials

**2.5.Database: Embase (via Ovid)**

Date Searched: 2025.9.10

Records Retrieved: 212

Search Query:

1. exp chronic kidney disease/

2. (chronic kidney disease or chronic renal disease or CKD or ESRD or ESKD).ti,ab,kw.

3. 1 or 2

4. exp mediterranean diet/

5. (mediterranean diet or mediterranean dietary pattern).ti,ab,kw.

6. 4 or 5

7. exp randomized controlled trial/

8. random*.ti,ab,kw.

9. clinical trial*.ti,ab,kw.

10. placebo.ti,ab,kw.

11. 7 or 8 or 9 or 10

12. exp cohort analysis/

13. (cohort or prospective or longitudinal or observational).ti,ab,kw.

14. 12 or 13

15. 11 or 14

16. 3 and 6 and 15

17. limit 16 to human

**2.6. ClinicalTrials.gov**

Date Searched: 2025.9.10

Records Retrieved: 13

Search Query:

Condition or disease: "chronic kidney disease" OR "renal insufficiency"

Other terms: "mediterranean diet"

Study type: All

Status: All

****2.7. Search Scope Clarification****
The search strategy was designed to be broad and sensitive to capture all potentially relevant literature on the Mediterranean diet and chronic kidney disease. **The search queries did NOT incorporate exclusion terms (e.g., dialysis, hemodialysis, kidney transplant) at the database search stage.** This approach was adopted to avoid prematurely excluding studies that might include mixed populations (e.g., studies covering a spectrum of CKD stages where non-dialysis patient data are reported separately) or studies where the patient population is not fully specified in the title or abstract but is detailed in the full text. The explicit exclusion of studies exclusively involving dialysis or kidney transplant recipients, as per the review's eligibility criteria (P: non-dialysis CKD patients), was performed during the subsequent **title/abstract screening** and **full-text review** phases by two independent reviewers.

3. Search Management

All records were imported into Zotero, version 7 for deduplication.

Duplicates were removed first by the software's automatic function, followed by a manual check.

The screening of titles/abstracts and full texts was conducted independently by two reviewers (ZC and YT).
