# Supplementary material for: Mediterranean diet with high-phenolic EVOO slows kidney function decline and reduces inflammation in nondialysis CKD: a meta-analysis
Source: Front Nutr. 2026 Mar 2;13:1792390. doi: 10.3389/fnut.2026.1792390 (PMC12989350; doi:10.3389/fnut.2026.1792390)
Supplement: Supplementary file 3 [file Table_3.docx]

This supplement provides the detailed formulas, calculations, and procedures referenced in the main manuscript’s Data Synthesis and Statistical Analysis section.

The included studies presented outcome data in varying formats. To integrate all available data, we employed the following strategy:

To address potential baseline imbalances and integrate studies reporting outcomes in different formats, we synthesized data using the mean difference (MD) in change from baseline as the unified effect measure.

For studies reporting means and standard deviations (SDs) at baseline and post-intervention, we calculated the MD and standard error (SE) of change scores. The SD of the change was imputed using the formula:

$$\text{SD}_{\text{c}\text{h}\text{ange}}\text{ = }\sqrt{\text{SD}_{\text{base}}^{\text{2}}\text{ + }\text{SD}_{\text{final}}^{\text{2}}\text{−2 }\text{×}\text{ }\text{r}\text{ }\text{×}\text{ }\text{SD}_{\text{base}\text{ }}\text{×}{\text{ }\text{SD}}_{\text{final}}}$$

We assumed a correlation coefficient (r) of 0.7 between timepoints—a value reflecting the moderate-to-high stability of cardiometabolic and renal markers in chronic conditions such as CKD^1,2^. For studies directly reporting the MD of change scores (with confidence intervals or standard errors), these values were used directly. The SE was calculated from 95% confidence intervals using a t-distribution appropriate for the study’s sample size $\text{df}\text{ = }\text{n}_{\text{int}}\text{ + }\text{n}_{\text{con}}\text{ − 2}$

All effect estimates were pooled using the generic inverse-variance method under a random-effects model as described by DerSimonian and Laird^13^. Heterogeneity was quantified using the I² statistic^14^, with values of 25%, 50%, and 75% representing low, moderate, and high heterogeneity, respectively. To assess the robustness of our primary analysis, we conducted sensitivity analyses by varying the imputation correlation coefficient (r = 0.4) and (r = 0.9).

**1. Categorization of Studies Based on Reported Data**

Studies were classified into three categories to standardize the calculation of the effect size (Mean Difference in Change from Baseline):

Category A:

Studies reporting means and standard deviations (SDs) at baseline and post-intervention for each group.

Calculation required: Imputation of change score variances using an assumed correlation between baseline and final values.

Category B:

Studies directly reporting the Mean Difference (MD) of change scores between groups, along with its 95% Confidence Interval (CI), Standard Error (SE), or standard deviations.

Calculation required: Use the reported MD and derive use its SE directly.

Category C:

Studies reporting the mean change and its standard deviation (SD) within each group.

Calculation required: Direct calculation of the MD and its SE from the group-level change data.

**2. Detailed Formulas for Effect Size Calculation**

For Category A Studies (Imputing Change Scores)

Step 1: Calculate the mean change for each group.

$$\text{Mean}_{\text{c}\text{h}\text{ange}}\text{ =}{\text{ }\text{Mean}}_{\text{final}\text{ }}\text{−}{\text{ }\text{Mean}}_{\text{base}}$$

Step 2: Calculate the Mean Difference (MD) in change between groups.

$$\text{MD}_{\text{change}\text{ }}\text{= }\text{MD}_{\text{change}\text{, }\text{int}\text{ }}\text{−}\text{MD}_{\text{change}\text{, }\text{con}\text{ }}$$

Step 3: Estimate the Standard Deviation (SD) of the change for each group using an assumed correlation coefficient (r) between baseline and final measurements.

$$\text{SD}_{\text{c}\text{h}\text{ange}}\text{ = }\sqrt{\text{SD}_{\text{base}}^{\text{2}}\text{ + }\text{SD}_{\text{final}}^{\text{2}}\text{−2 }\text{×}\text{ }\text{r}\text{ }\text{×}\text{ }\text{SD}_{\text{base}\text{ }}\text{×}{\text{ }\text{SD}}_{\text{final}}}$$

Rationale for (r = 0.7): This value is based on the moderate-to-high stability of core renal (e.g., eGFR, creatinine) and metabolic (e.g., LDL-C, systolic blood pressure) biomarkers over typical trial durations in CKD populations, as supported by longitudinal cohort data and methodological guidelines for meta-analysis^1,2^.

Step 4: Calculate the Standard Error (SE) of ($\text{MD}_{\text{change}\text{ }}$).

$$\text{SE}_{\text{c}\text{h}\text{ange}\text{ }}\text{= }\sqrt{\frac{\text{SD}_{\text{c}\text{h}\text{ange}\text{, }\text{int}}^{\text{2}}}{\text{n}_{\text{int}}}\text{ + }\frac{\text{SD}_{\text{c}\text{h}\text{ange}\text{, }\text{con}}^{\text{2}}}{\text{n}_{\text{con}}}}$$

For Category B Studies (Using Directly Reported Change Score MDs)

If the Standard Error (SE) is reported directly, it is used.

If a 95% Confidence Interval (CI) is reported, the SE is calculated using the t-distribution:

$$\text{SE}\text{ = }\frac{\text{Upper}_{\text{95\%}\text{CI}\text{ }}\text{−}{\text{ }\text{Lower}}_{\text{95\%}\text{CI}}}{\text{2 }\text{×}{\text{ }\text{t}}_{\text{α}\text{/2，}\text{df}}}$$

where (**t)** is the critical value from the two-tailed t-distribution for a 95% confidence level with degrees of freedom $\text{df}\text{ = }\text{n}_{\text{int}}\text{ + }\text{n}_{\text{con}}\text{ − 2}$

If only the SDs of the change for each group are reported, the SE is calculated as in Step 4 for Category A.

For Category C Studies

For studies that directly report the mean change and its standard deviation (SD_change_) within each group:

Step 1: The Mean Difference (MD) in change between groups is:

$$\text{MD}_{\text{change}\text{ }}\text{= }\text{MD}_{\text{change}\text{, }\text{int}\text{ }}\text{−}\text{MD}_{\text{change}\text{, }\text{con}\text{ }}$$

Step 2: The Standard Error (SE) of this MD is calculated as:

$$\text{SE}_{\text{c}\text{h}\text{ange}\text{ }}\text{= }\sqrt{\frac{\text{SD}_{\text{c}\text{h}\text{ange}\text{, }\text{int}}^{\text{2}}}{\text{n}_{\text{int}}}\text{ + }\frac{\text{SD}_{\text{c}\text{h}\text{ange}\text{, }\text{con}}^{\text{2}}}{\text{n}_{\text{con}}}}$$

However, some studies report medians with ranges (minimum–maximum) or interquartile ranges (IQRs), particularly for non-normally distributed outcomes or small sample sizes.To include these studies in our quantitative synthesis, we applied validated statistical methods to estimate means and SDs from reported medians and ranges.

We used the method developed by Wan et al. (2014)^3^, which provides formulas to estimate the sample mean and SD from the sample size, median, and range. This method was selected because:- It is specifically designed for the common scenario where only median, range, and sample size are available- It includes a small-sample correction term for mean estimation- It has been validated through extensive simulation studies showing minimal bias- It performs well across various sample sizes and distribution shapes.

The complete Wan et al. formulas are:

Mean estimation:

$$Mean=\frac{a+2m+b}{4}+\frac{a-2m+b}{4n}$$

Where $a$=minimum, $m$=median, $b$=maximum, $n$=sample size.

Standard deviation estimation:

$$SD=\frac{b-a}{{2\Phi}^{-1}\left( \frac{n-0.375}{n+0.25} \right)}$$

3. Data Synthesis and Sensitivity Analysis Protocol

**Pooling Procedure**

All calculated (MD_change_) and (SE_change_) values were synthesized using the Generic Inverse-Variance method in RevMan 5.4, under a random-effects model (DerSimonian and Laird).

**Heterogeneity Assessment**

Statistical heterogeneity was quantified using the I² statistic.

**Sensitivity Analyses on Imputation Assumption**

To evaluate the robustness of our imputation approach, we conducted the following sensitivity analyses:

1. Primary Analysis: Assumed (r = 0.7) for all Category A studies.

2. Sensitivity Analysis 1: Repeated pooling assuming a lower correlation (r = 0.4).

3. Sensitivity Analysis 2: Repeated pooling assuming a higher correlation (r = 0.9).

The consistency of the pooled effect estimate and its statistical significance across these scenarios was used to assess the robustness of our conclusions.

**References for Supplementary Material S2:**

1. F. Tentori et al., Stability of cardiovascular and renal biomarkers in chronic kidney disease: a longitudinal analysis from the CRIC study, American Journal of Kidney Diseases, vol. 65, no. 2, pp. 267–275, 2015.
2. J. P. T. Higgins et al., Cochrane Handbook for Systematic Reviews of Interventions, Version 6.4, 2023. Section 6.5.2.8 – Imputing standard deviations for changes from baseline.
3. Wan X, Wang W, Liu J, Tong T. Estimating the sample mean and standarddeviation from the sample size, median, range and/or interquartile range. *BMC Medical Research Methodology*. 2014;14:135. doi:10.1186/1471-2288-14-135
4. R. DerSimonian & N. Laird, Meta-analysis in clinical trials, Control Clin Trials, vol. 7, no. 3, pp. 177–188, 1986.
5. J. P. T. Higgins et al., Measuring inconsistency in meta-analyses, BMJ, vol. 327, no. 7414, pp. 557–560, 2003.
