# Supplementary material for: Mediterranean diet with high-phenolic EVOO slows kidney function decline and reduces inflammation in nondialysis CKD: a meta-analysis
Source: Front Nutr. 2026 Mar 2;13:1792390. doi: 10.3389/fnut.2026.1792390 (PMC12989350; doi:10.3389/fnut.2026.1792390)
Supplement: Supplementary file 4 [file Table_4.docx]

**Introduction:**

This supplementary file presents the forest plots from sensitivity analyses performed to assess the robustness of our primary meta-analysis results to the assumed correlation coefficient (r) between baseline and follow-up measurements. Theprimary analysis assumed r=0.7. Here we show results under lower (r=0.4) andhigher (r=0.9) correlation assumptions.

1. **Renal Function and Safety Outcomes**
   1. ****Kidney Function (eGFR)****

**(r=0.4)**

**
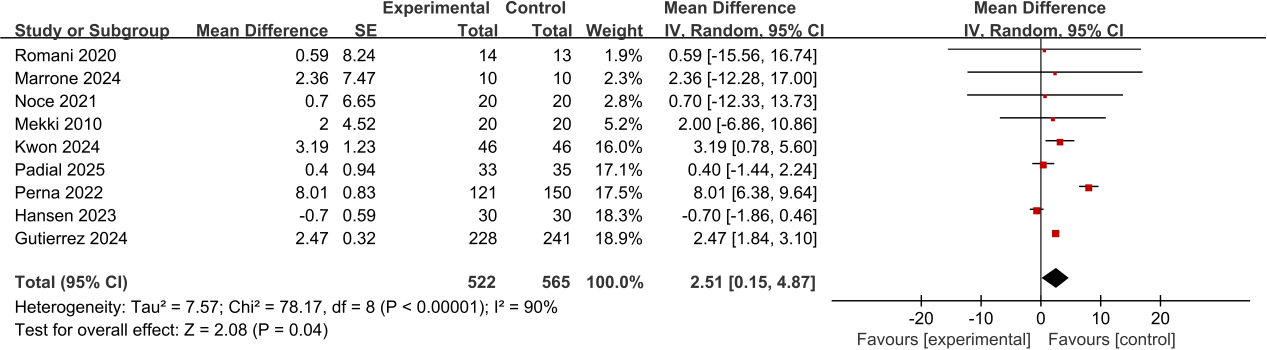
**

**(r=0.9)**

**
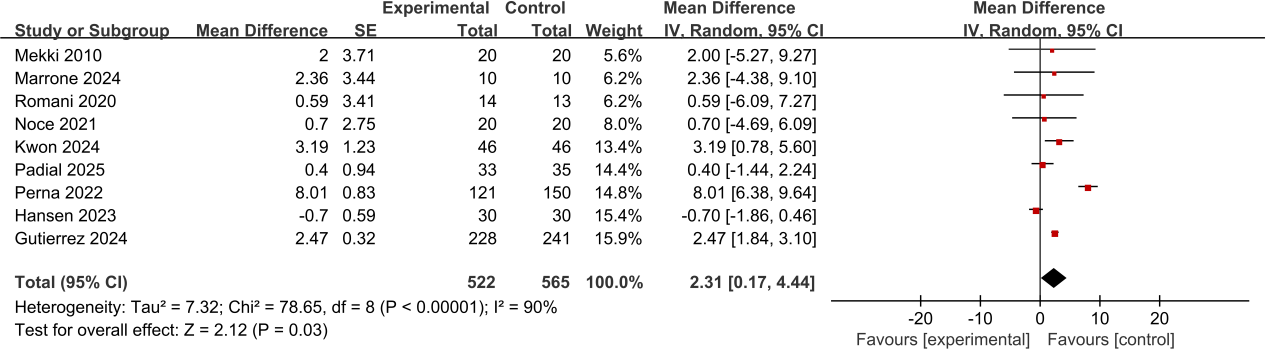
**

- 1. **Serum Creatinine**

**(r=0.4)**

**
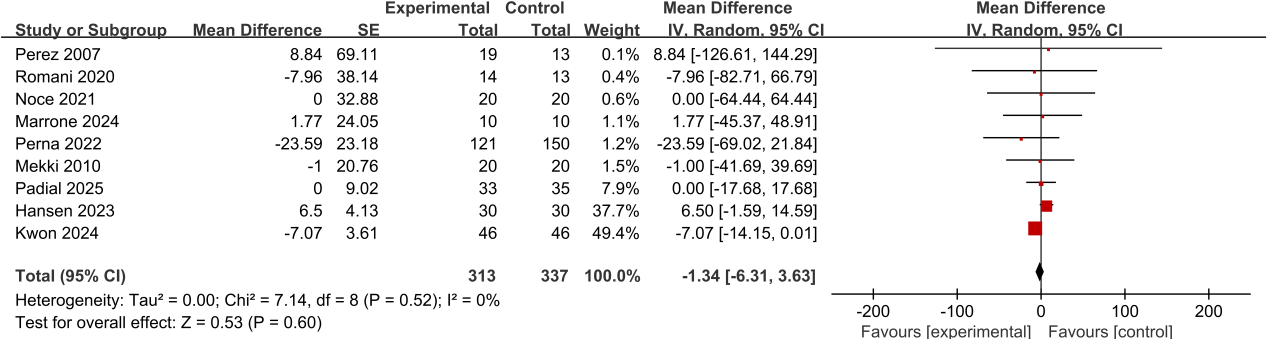
**

**(r=0.9)**

**
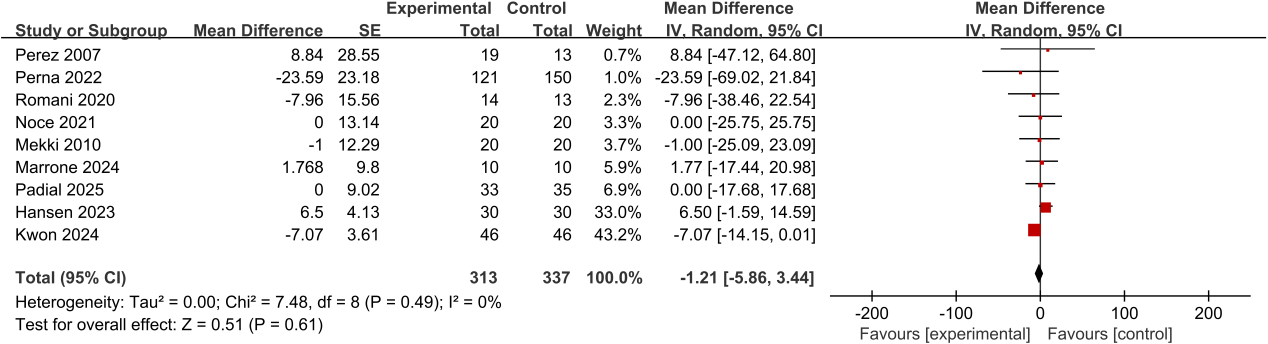
**

- 1. **Blood Urea Nitrogen(BUN)**

**(r=0.4)**

**
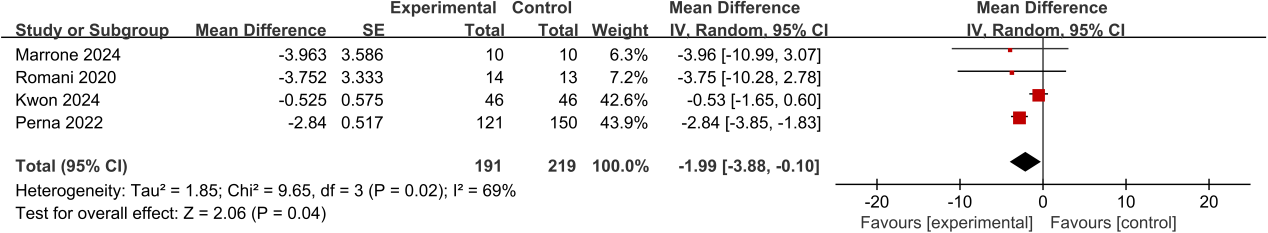
**

**(r=0.9)**

**
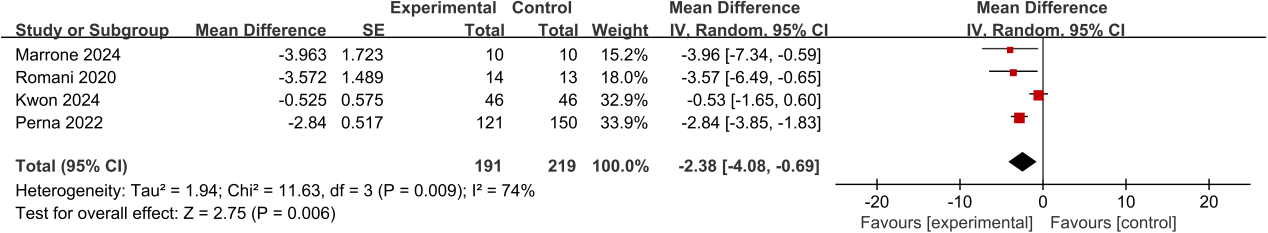
**

**1.4 Serum Potassium**

**(r=0.4)**

**
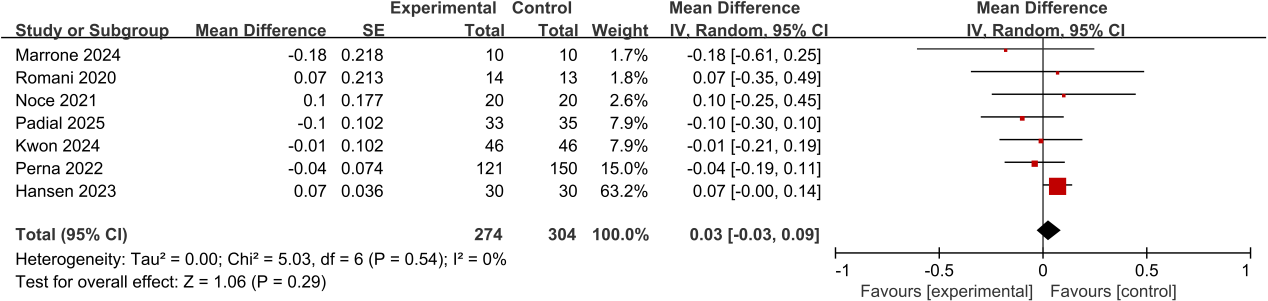
**

**(r=0.9)**

**
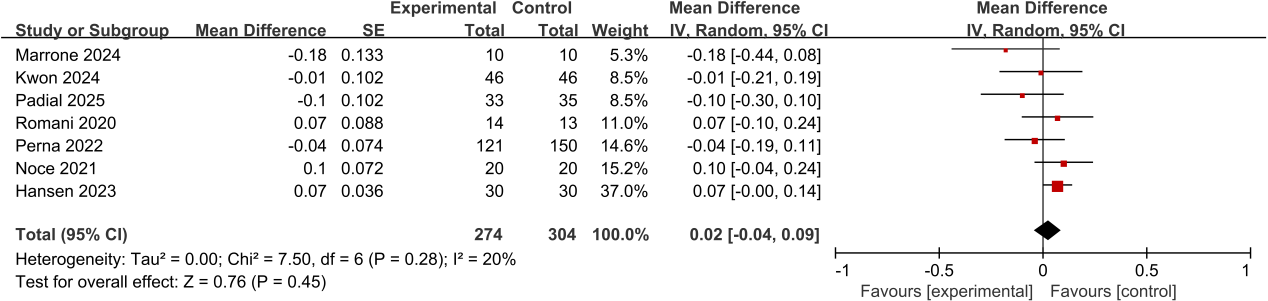
**

**1.5 Serum Phosphorus**

**(r=0.4)**

**
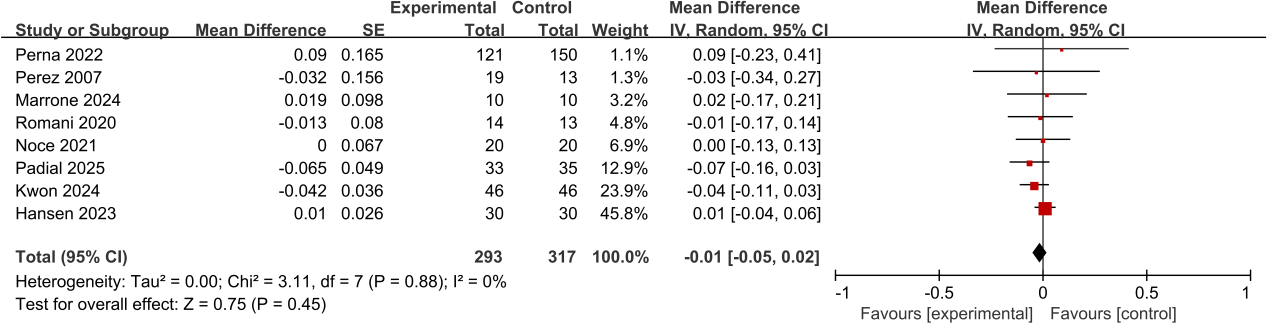
**

**(r=0.9)**

**
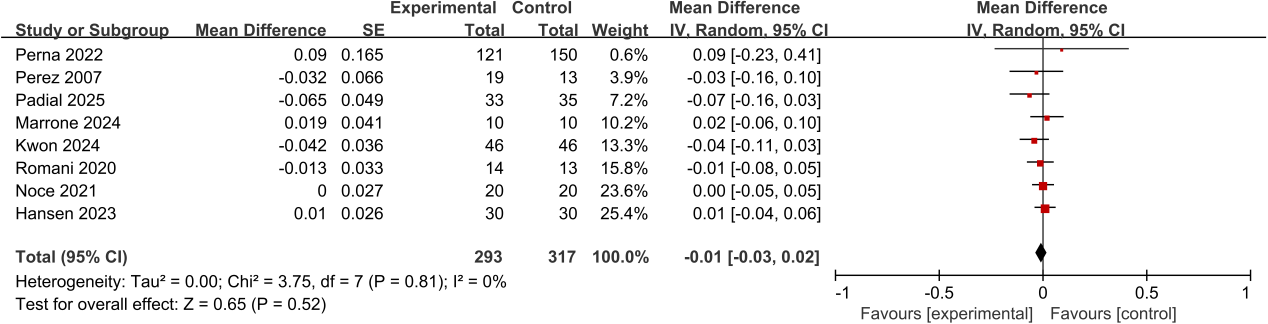
**

1. **Cardiometabolic Outcomes**

**2.1 Lipid Profiles**

**2.1.1 Triglyceride (r=0.4 & r=0.9)**

**
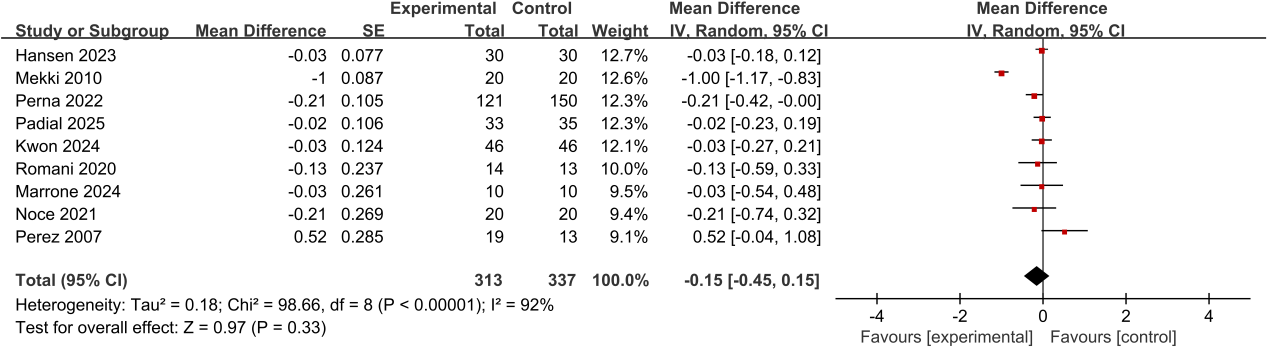
**

**
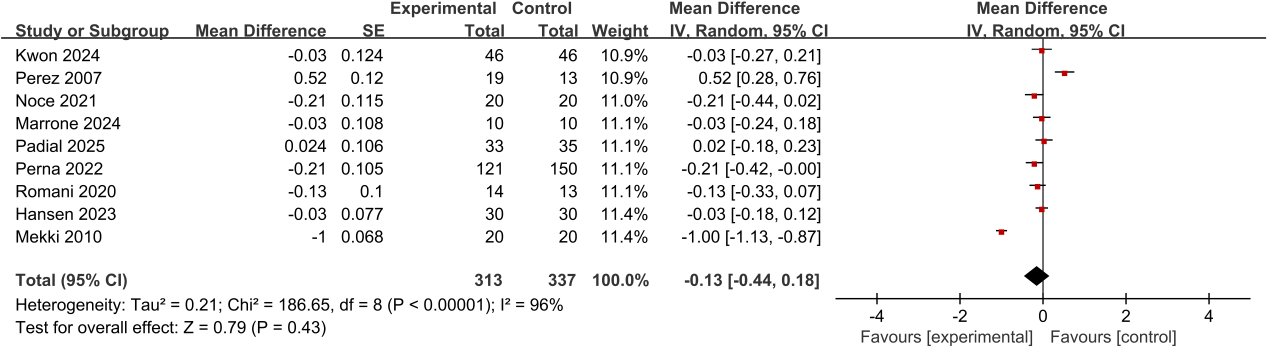
2.1.2 Total Cholesterol (r=0.4 & r=0.9)**

**
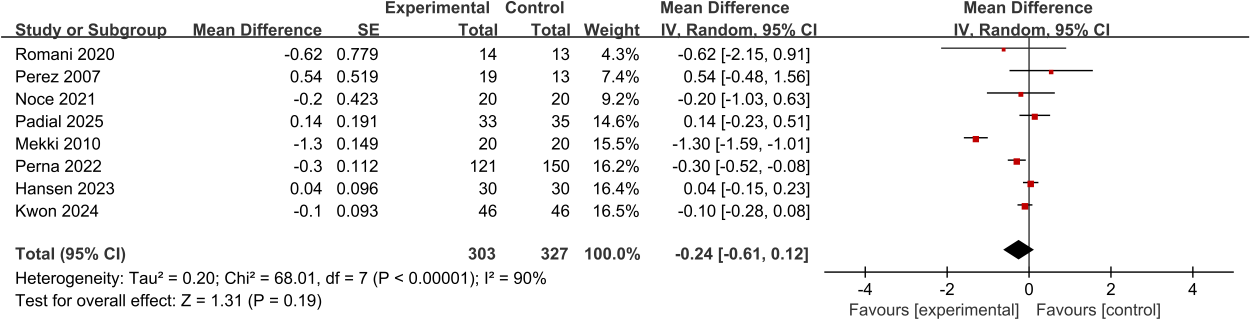
**

**
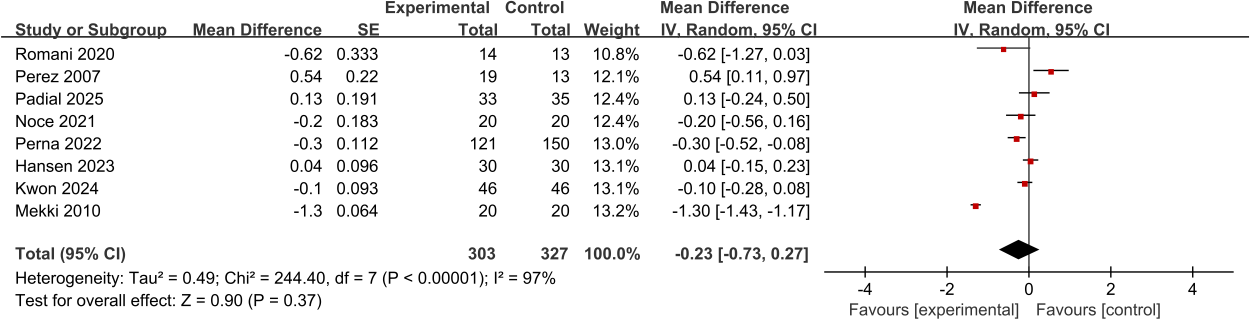
**

****2.1.3 High-Density Lipoprotein Cholesterol (HDL-C)** (r=0.4 & r=0.9)**

**
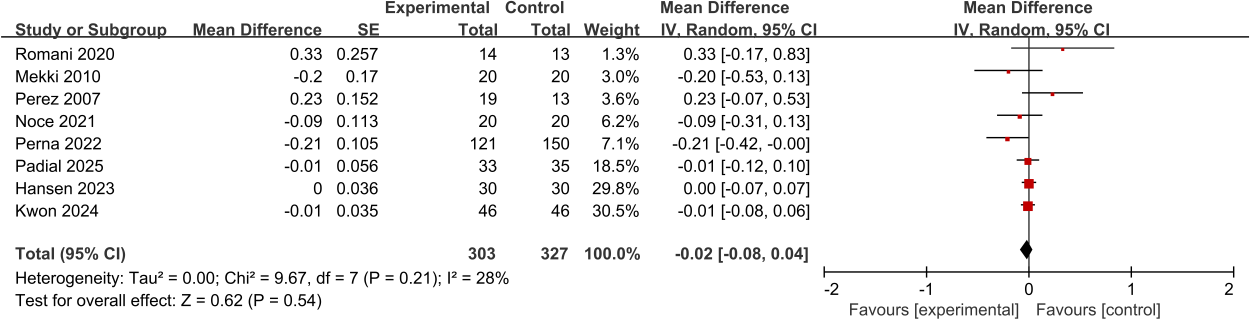
**

**
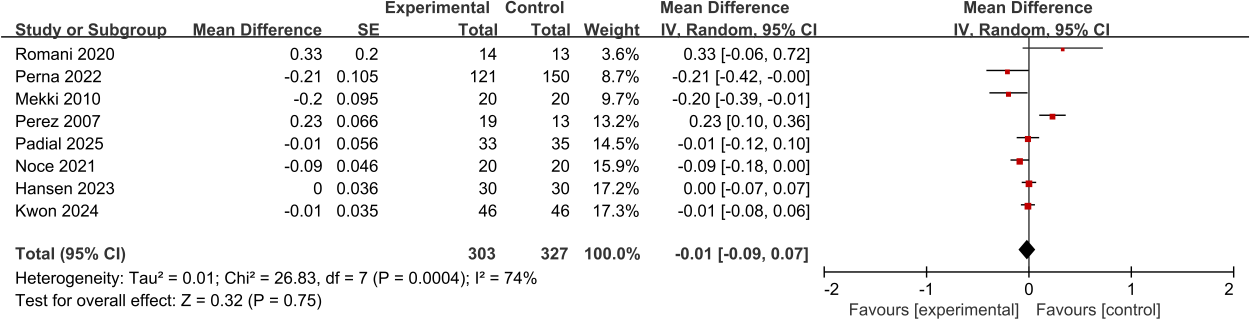
**

**2.1.4 **Low-Density Lipoprotein Cholesterol (LDL-C)** (r=0.4 & r=0.9)**

**
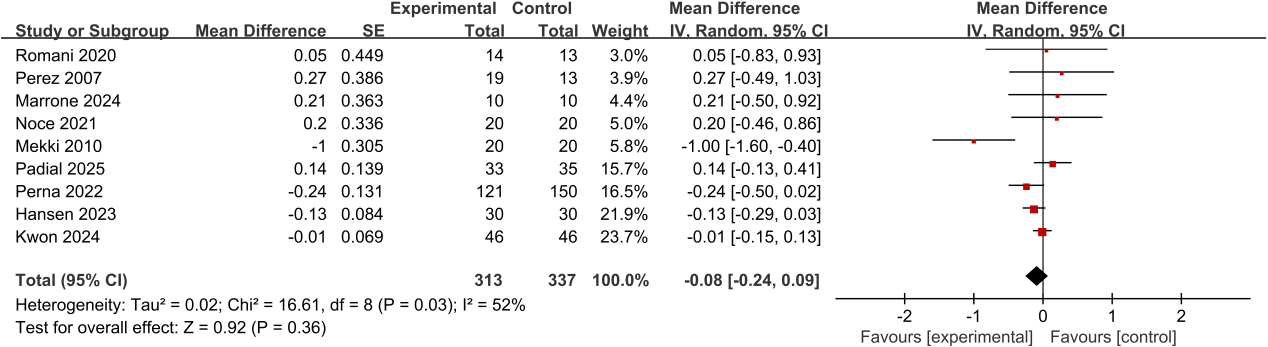
**

**
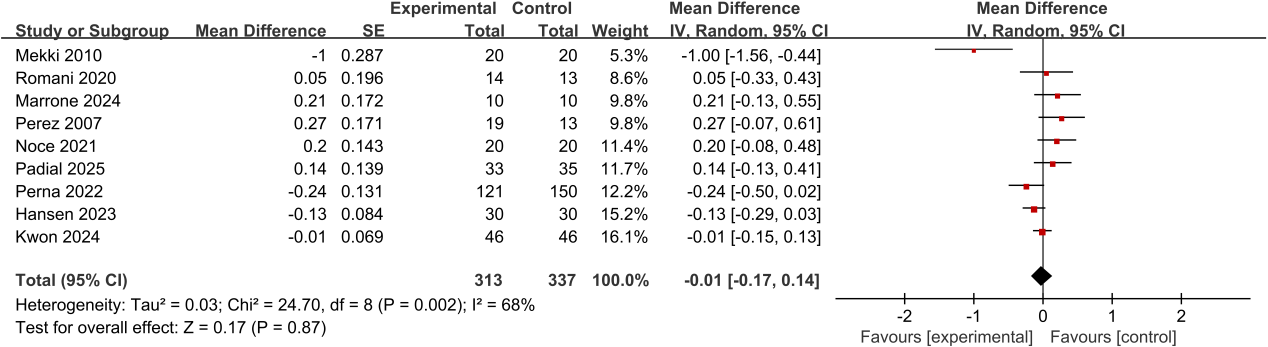
**

**2.2 Blood Pressure**

**2.2.1 Systolic Blood Pressure (SBP) (r=0.4 & r=0.9)**

**
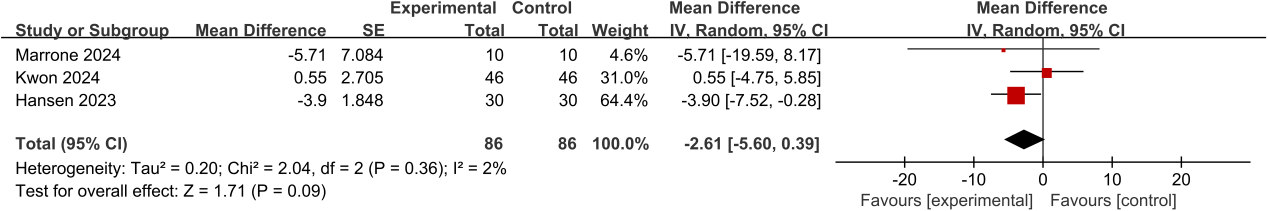
**

**
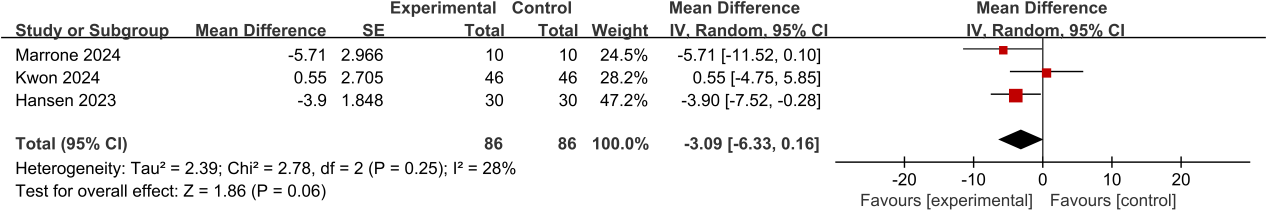
**

**2.2.2 Diastolic Blood Pressure (DBP) (r=0.4 & r=0.9)**

**
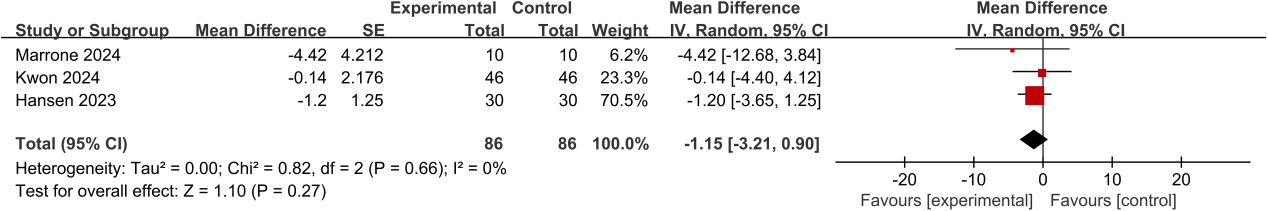

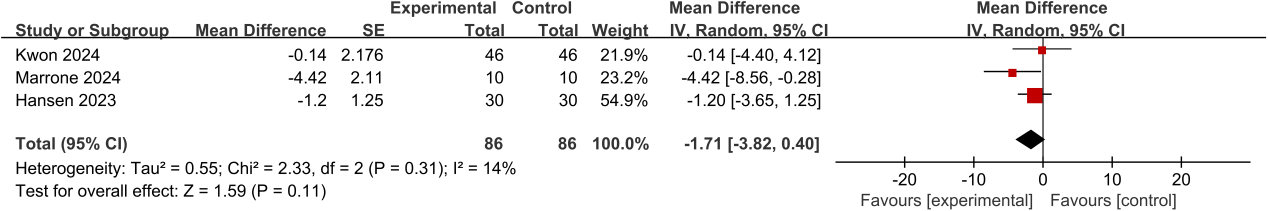
**

**2.3 Fasting Blood Glucose (FBG) (r=0.4 & r=0.9)**

**
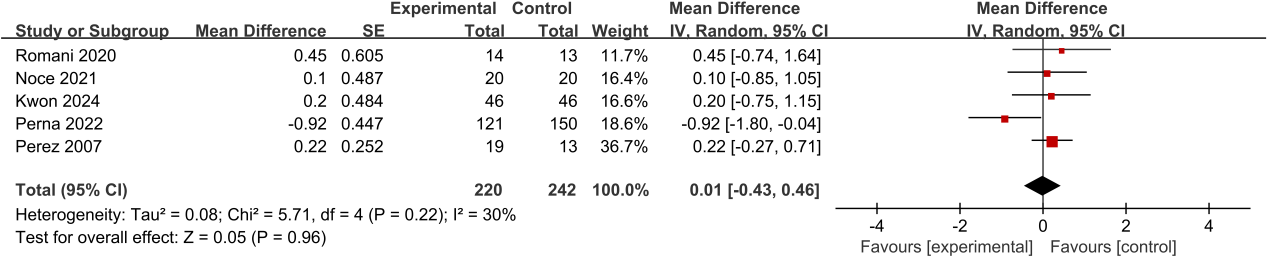
**

**
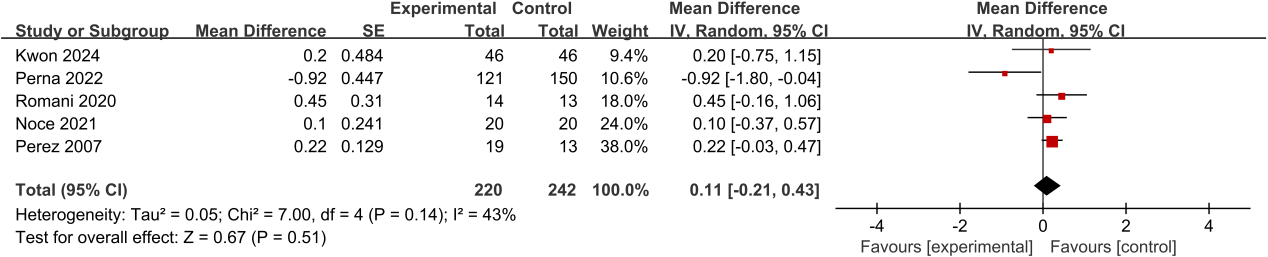
**

**3. Body Composition Outcomes**

**3.1 Body Weight (r=0.4 & r=0.9)**

**
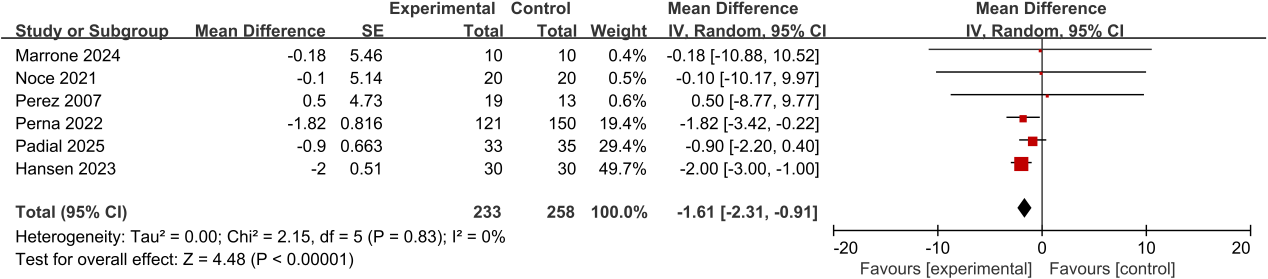
**

**
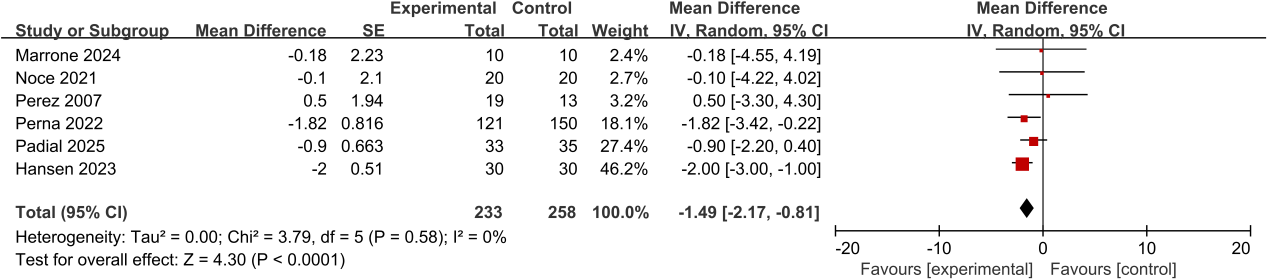
**

****3.2 Body Mass Index (BMI)** (r=0.4 & r=0.9)**

**
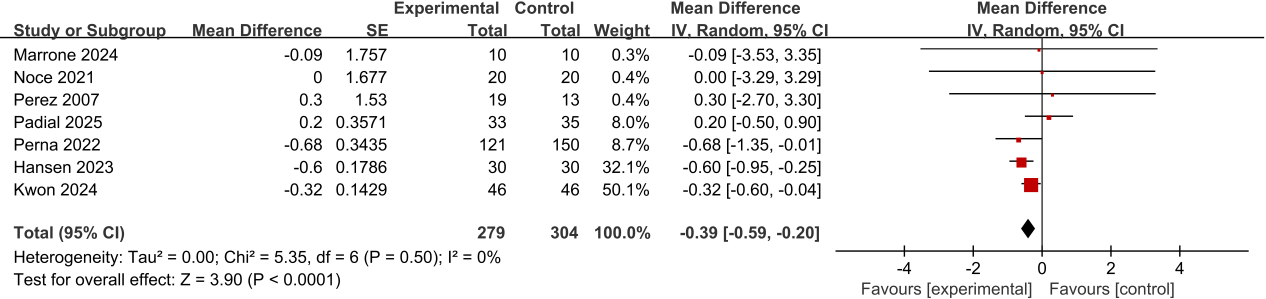
**

**
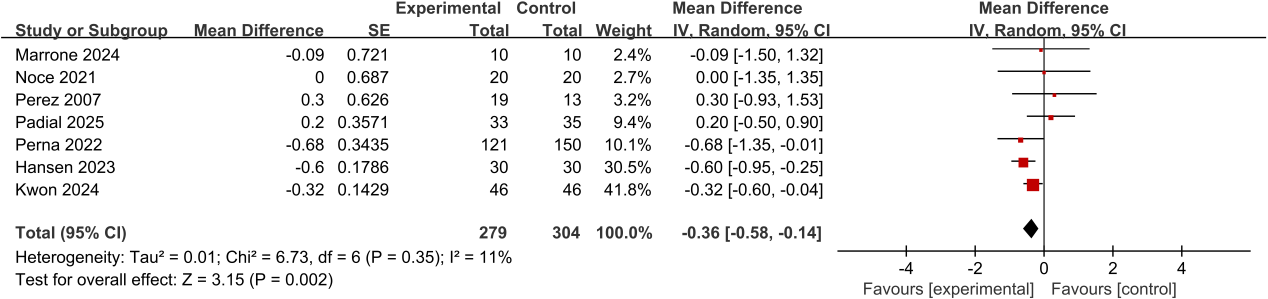
**

****3.3 Fat Mass (FM)** (r=0.4 & r=0.9)**

**
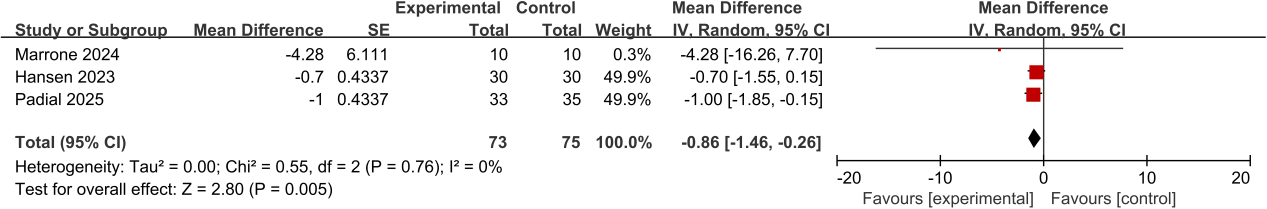
**

**
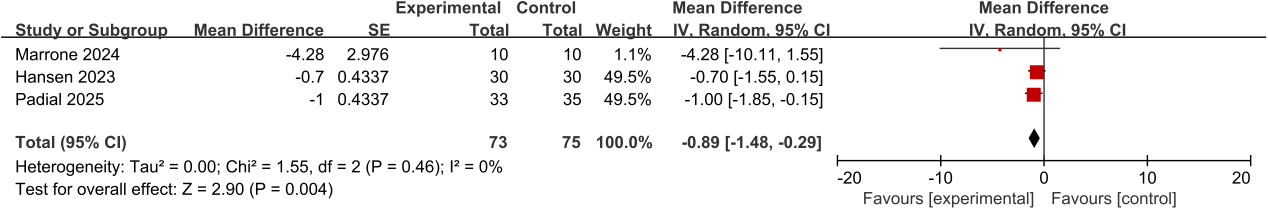
**

**4. Nutritional and Inflammatory Markers**

****4.1 Serum Albumin** (r=0.4 & r=0.9)**

**
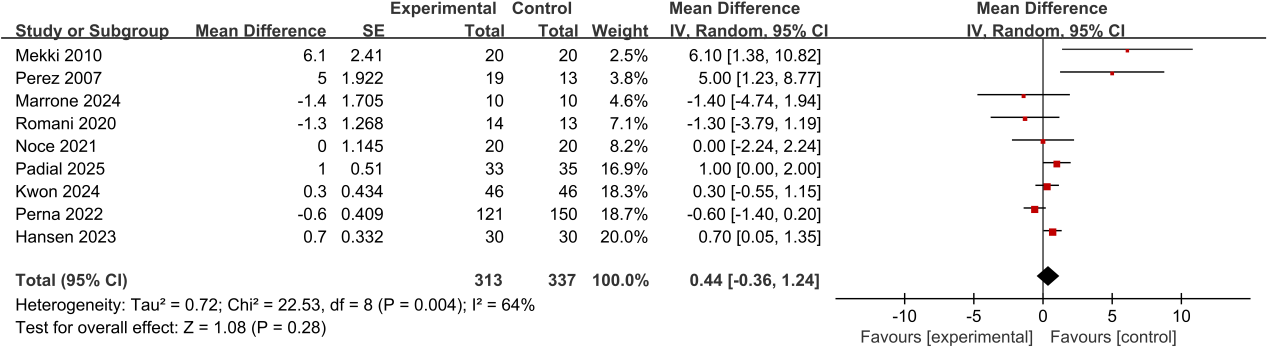
**

**
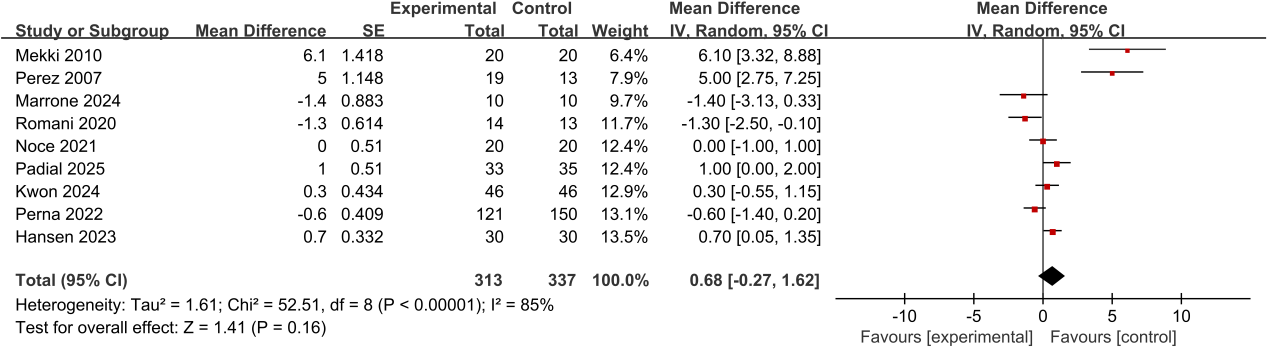
**

****4.2 Hemoglobin** (r=0.4 & r=0.9)**

**
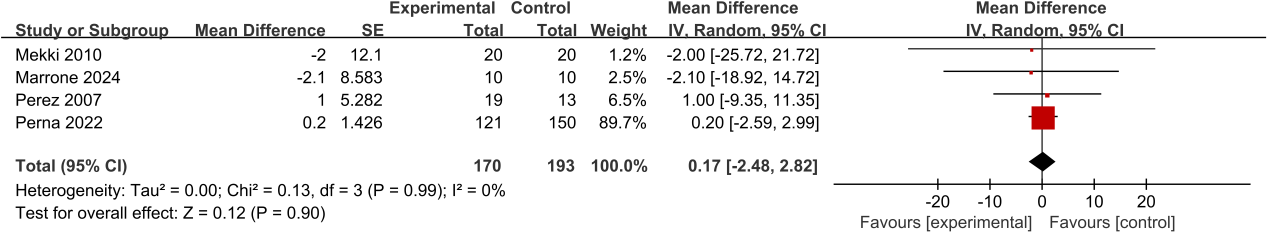
**

**
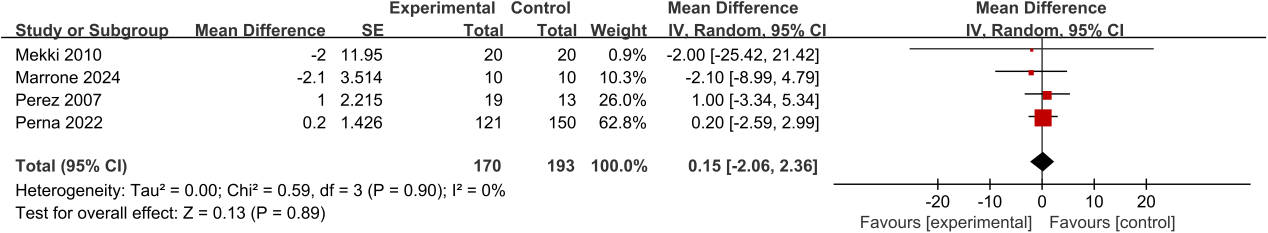
**

**4.3 C-**Reactive Protein (CRP)** (r=0.4 & r=0.9)**

**
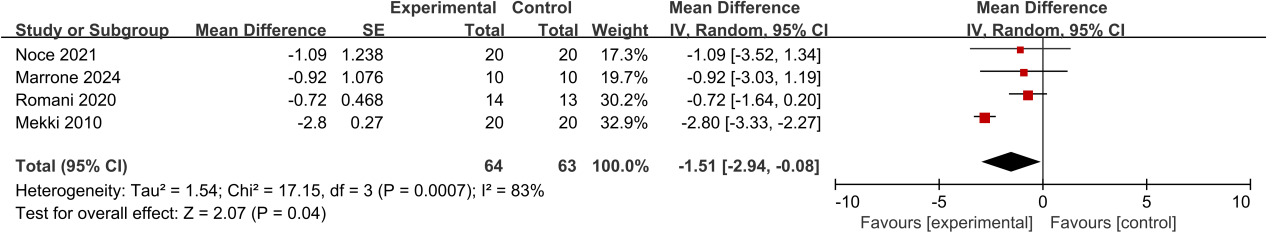
**

**
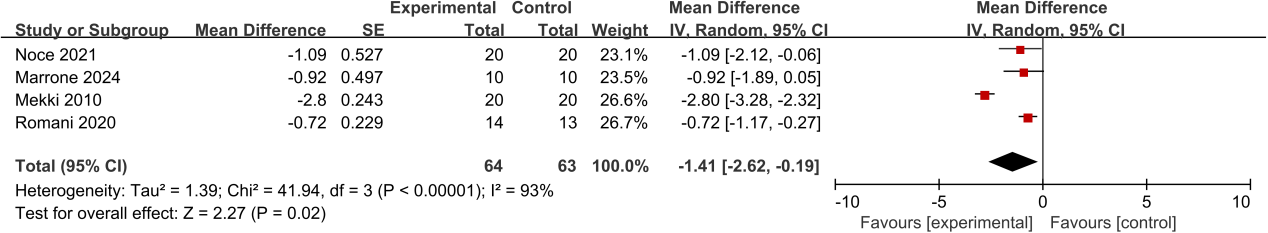
**
